# A Two-Phase Innate Host Response to Alphavirus Infection Identified by mRNP-Tagging In Vivo

**DOI:** 10.1371/journal.ppat.0030199

**Published:** 2007-12-21

**Authors:** Jennifer L Konopka, Luiz O Penalva, Joseph M Thompson, Laura J White, Clayton W Beard, Jack D Keene, Robert E Johnston

**Affiliations:** 1 Department of Microbiology and Immunology University of North Carolina at Chapel Hill, Chapel Hill, North Carolina, United States of America; 2 Carolina Vaccine Institute, University of North Carolina at Chapel Hill, Chapel Hill, North Carolina, United States of America; 3 Department of Molecular Genetics and Microbiology, Center for RNA Biology, Duke University Medical Center, Durham, North Carolina, United States of America; The Rockefeller University, United States of America

## Abstract

A concept fundamental to viral pathogenesis is that infection induces specific changes within the host cell, within specific tissues, or within the entire animal. These changes are reflected in a cascade of altered transcription patterns evident during infection. However, elucidation of this cascade *in vivo* has been limited by a general inability to distinguish changes occurring in the minority of infected cells from those in surrounding uninfected cells. To circumvent this inherent limitation of traditional gene expression profiling methods, an innovative mRNP-tagging technique was implemented to isolate host mRNA specifically from infected cells *in vitro* as well as *in vivo* following Venezuelan equine encephalitis virus (VEE) infection. This technique facilitated a direct characterization of the host defense response specifically within the first cells infected with VEE, while simultaneous total RNA analysis assessed the collective response of both the infected and uninfected cells. The result was a unique, multifaceted profile of the early response to VEE infection in primary dendritic cells, as well as in the draining lymph node, the initially targeted tissue in the mouse model. A dynamic environment of complex interactions was revealed, and suggested a two-step innate response in which activation of a subset of host genes in infected cells subsequently leads to activation of the surrounding uninfected cells. Our findings suggest that the application of viral mRNP-tagging systems, as introduced here, will facilitate a much more detailed understanding of the highly coordinated host response to infectious agents.

## Author Summary

A major element of viral pathogenesis is the induction of specific changes within the infected host, often reflected in altered gene expression patterns. However, revealing these changes *in vivo* has been limited by an inability to distinguish changes within the minority of infected cells from that in surrounding uninfected cells. Here we introduce a viral mRNP-tagging system, based on Venezuelan equine encephalitis virus (VEE), that enables the isolation of host mRNA specifically from infected cells *in vitro* and *in vivo*, even when they are a small minority. This system allowed us for the first time to monitor the innate response specifically within the cells initially infected *in vivo*. In combination with simultaneous analysis of the entire tissue response, the result was a multifaceted view of the innate response to VEE in dendritic cells, and in the draining lymph node. The results supported a two-step response in which activation of host genes within infected cells leads to activation of bystander cells, offering insight into the process by which the greater innate immune response to alphaviruses is established *in vivo*. This system may be employed for a wide variety of pathogens, offering broader implications to the manner in which interactions between pathogens and their hosts are studied.

## Introduction

At the interface of pathogen infection and host response lies a complex network of regulated interactions. As the host seeks to eradicate the pathogen and maintain survival, the pathogen itself seeks to continue its own proliferation at whatever cost is necessary to the host cell.

Therefore, the insult associated with viral infection often involves numerous changes in host gene expression. Fundamental to many viral pathogenesis studies is the investigation of these specific changes within the host cell, or on a more global scale, within a specific tissue, organ, or the entire animal. Although it has been possible in several systems to singularly identify cellular genes that are altered in expression due to infection, these genes most likely represent a very small fraction of all the genes induced or repressed. In an attempt to more fully understand the interactions between pathogen and host, virologists have turned to high-throughput genomic profiling technologies within the past decade to evaluate the status of host gene expression post-infection. Although widely informative, there remains an inherent limitation in applying these analyses to viral pathogenesis studies. In the absence of an acutely susceptible system in which all cells can be uniformly infected, a heterogeneous environment of infected and uninfected cells naturally exists during viral infection. This is particularly true *in vivo*, where only a minority of cells in a given tissue or organ are infected, even when that tissue is a major target of infection. In traditional gene expression analysis utilizing total RNA isolation, there is an inability to discriminate the population of host mRNAs isolated from the infected cells versus the surrounding uninfected cells. As the percentage of uninfected cells is high *in vivo*, mRNA from uninfected cells likely creates background signal that skews or masks the analysis from infected cells. Discriminating the direct viral impact on infected cells from the subsequent effects on bystander uninfected cells is critical to fully understanding the pathogenesis of a given virus, yet most analyses lack this distinction.

To circumvent this limitation, we have optimized and implemented an innovative mRNP-tagging technique to isolate host mRNA specifically from infected cells following viral infection in cultured cells as well as tissues *in vivo*. The mRNP-tagging technology was originally developed from a functional genomics approach termed *ribonomics*, which examines mRNAs functionally clustered in ribonucleoprotein complexes [1,2]. The mRNP-tagging system takes advantage of the natural interaction of RNA-binding proteins with cellular mRNA to effectively enrich and isolate messages from a specific minority cell type within a heterogeneous environment. One such interaction that has been used in several systems is the well established strong binding of poly(A) binding protein I (PABP) to the poly(A) tail of cellular mRNAs prior to translation [3–8]. In the mRNP-tagging technique, a unique version of PABP engineered with an epitope tag is expressed in a cell- or tissue-specific manner. The cellular mRNA bound to the tagged-PABP is then co-immunoprecipitated using an anti-epitope antibody, enriching the mRNA from the targeted cell population and separating it from the mRNA of the surrounding cells or tissues [1,2,9]. Gene profiling methods such as cDNA microarrays or quantitative real-time PCR can then be performed using the enriched mRNA population to assess the gene expression status within the cell or tissue population of interest. This method has been successfully applied to identify tissue-specific mRNA populations in Caenohabditis elegans and *Drosophila melanogaster* [10–13], as well as to identify cell type-specific gene expression changes in mixed cell culture models *in vitro* [14].

Here, we have adapted the mRNP-tagging technique to characterize host gene expression changes following infection with Venezuelan equine encephalitis virus (VEE). VEE is an arthropod-borne, single-stranded (+)sense RNA virus associated with periodic epidemics and equine epizootics in the Western Hemisphere, and serves as a leading model for the study of alphavirus pathogenesis [15]. Numerous studies have underscored the dramatic role of virus genetics and the subsequent host defense response in dictating the course and outcome of VEE infection [16–32]. Although infection in the murine model has been well studied for some time, little is known concerning the molecular markers of VEE-induced disease, including the direct effects on host cell gene expression. VEE infection is characterized by two distinct disease phases following infection in humans, horses, and mice: An initial lymphotropic phase characterized by a high serum viremia, followed by invasion of the central nervous system and initiation of a neurotropic phase leading to encephalitis. In horses and mice, progression to the neurotropic phase occurs at very high frequency. Previous studies in our laboratory have carefully examined the progression of pathogenesis in the mouse model, utilizing molecularly cloned infectious VEE as well as an extensive panel of mutants blocked at various stages of infection [18,21,22,25,27,29]. The draining lymph node (DLN), and in particular the dendritic cells, was subsequently identified as the initial site of viral replication, with infected Langerhans cells migrating there from the site of inoculation in the footpad [20]. It has been hypothesized that the early events within the DLN set the stage for the VEE-specific pattern of virus replication and host response. However, many details of the earliest stages of VEE infection remain largely undefined, with the innate host response likely playing a major role.

To define the molecular profile of the early virus–host interactions central to VEE pathogenesis, we took advantage of several tools. One tool paramount to studying the early events in infection are VEE replicon particles (VRP). VRP are propagation-defective vector particles that undergo only one round of infection, as the structural genes which normally drive the assembly of progeny virions are deleted and replaced with a marker gene of interest [33]. As such, VRP replication is limited to the first infected cells, allowing us to model the earliest events of VEE infection. In addition, the application of an mRNP-tagging technology offers an opportunity for a distinct view of the VEE-induced changes in host gene expression. By expressing an epitope-tagged version of PABP from VRP, host messages induced specifically within the first round of infected cells can be fractionated from those of the surrounding uninfected cells. Through co-immunoprecipitation with antibody to the epitope tag, the infected cell host mRNA bound to the VRP-delivered tagged-PABP can be isolated and screened as a discrete mRNA population for changes in host gene expression. This technology enables discrimination of uninfected cells from infected cells, and specifically profiles the changes induced in the infected cell population—a distinction that previously has been difficult to achieve, particularly *in vivo* where the infected cells may be only a small minority in a given tissue (e.g., in the DLN post-VRP infection).

Using VRP to infect primary dendritic cells *in vitro*, and to limit infection to the initially infected cells *in vivo*, we have elucidated gene expression patterns that define the early stages of VEE pathogenesis, including members of the interferon (IFN) and proinflammatory host defense pathways. This analysis revealed multifactorial interactions that occur within the virus-infected host, and indicated a two-phase innate response characterized by cytokine and antiviral gene induction profiles in the first infected cells that were distinct from that of uninfected bystander cells within the same tissue. By elucidating these specific and distinct host responses, systems such as the VRP mRNP-tagging approach have the potential to further our understanding of the complex interactions between pathogens and their hosts.

## Results

### Establishment of the VRP mRNP-Tagging System

**Figure 1:**
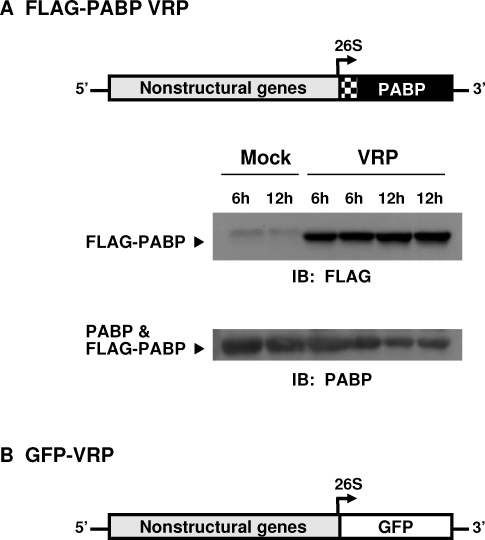
VEE Replicon Constructs The VEE replicon genome encodes the viral nonstructural genes, the authentic 5′ and 3′ UTR, as well as a cloning cassette downstream of the 26S subgenomic promoter for transgene insertion. Schematic representation of the replicon constructs used in this study are shown. (A) FLAG-PABP VRP, expressing an N-terminally FLAG-tagged version of PABP (FLAG epitope denoted by checkered shading). Expression of FLAG-PABP expressed from the VRP, as well as endogenous PABP, is shown by western blotting against the FLAG epitope or against PABP itself, from L929 cell lysates harvested at 6 and 12 hpi. (B) Schematic of GFP-VRP, expressing green fluorescent protein. All replicon particles used in this study were packaged in the wildtype (V3000) envelope.

An mRNP-tagging technique has been optimized and applied to isolate mRNA specifically from the infected cell population following VRP infection. The fundamental basis of mRNP-tagging relies on the natural interaction of RNA-binding proteins with RNA, with the synthesis of a uniquely tagged RNA binding protein in target cells enabling the isolation of the specific mRNA population through tag-specific antibodies and co-immunoprecipitation. To apply this technique to a virus infection model, a FLAG epitope-tagged version of PABP was delivered specifically to infected cells by engineering the virus itself to express the unique RNA-binding protein. The *PABP* coding sequence, with the FLAG epitope fused in frame at the 5′ end, was cloned directly downstream of the 26S promoter of the VEE replicon plasmid (Figure 1A), and the replicon RNA was packaged into VEE replicon particles to generate FLAG-PABP VRP. Upon infection of BHK and L929 cells, the FLAG-PABP VRP programmed the robust expression of the epitope-tagged version of PABP, as determined by anti-FLAG immunoprecipitation as well as by western blotting and from cell lysates (Figure 1A). Robust levels of expression were expected based on the documented high level of transgene expression from the 26S mRNA promoter of VRP [33], a key element for this VRP mRNP-tagging system.

**Figure 2:**
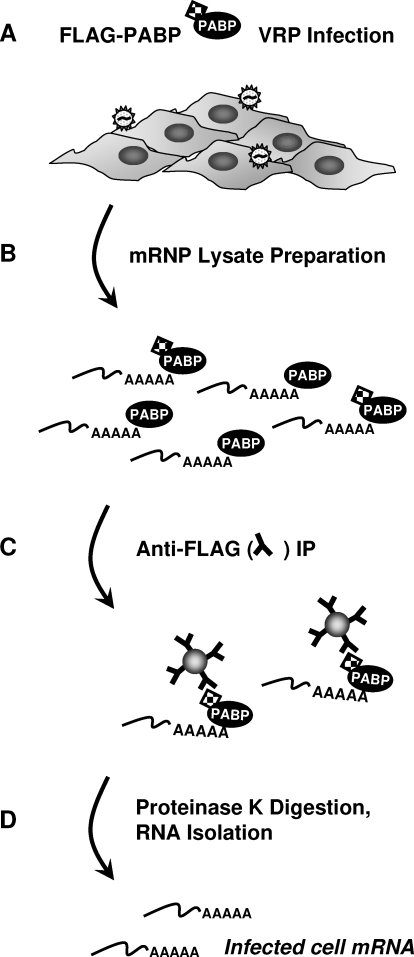
The VRP mRNP-Tagging System Flowchart illustrating the VRP mRNA-tagging method of isolating mRNA specifically from infected cells. (A) Cells are infected with FLAG-PABP VRP, delivering the unique epitope-tagged version of PABP only to infected cells. (B) At various times post-infection, cytoplasmic lysates are prepared, containing the cellular mRNP complexes. (C) Anti-FLAG antibody–coated agarose beads are added in excess to the lysate, co-immunoprecipitating the mRNA bound by FLAG-PABP, and thus fractionating the mRNA in the infected cells from the mRNA in the surrounding uninfected cells. (D) The immunoprecipitated mRNA-PABP complex is dissociated using proteinase K digestion, and the infected cell mRNA is isolated by standard RNA extraction and precipitation.

An outline of the VRP mRNP-tagging procedure is shown in Figure 2. Briefly, following infection with FLAG-PABP VRP at low multiplicity of infection (MOI), the FLAG-tagged PABP molecule is synthesized only in the infected cells as the replicon RNA is expressed. At various times post-infection the cells are lysed, releasing PABP-bound messages. Pre-cleared lysate is mixed with agarose beads coated with anti-FLAG antibody. The mRNA from the infected cells is immunoprecipitated specifically via the FLAG epitope of the PABP bound to the message in the RNP complex, as this form of PABP is only present in VRP-infected cells. The message is subsequently isolated from that complex by proteinase K digestion and phenol-chloroform based extraction. RNase protection assays (RPAs) were initially utilized to detect host messages following the anti-FLAG immunoprecipitation from infected L929 cell extracts, and verified that the VRP-supplied tagged-PABP was in fact bound to host messages, and the host mRNA could be isolated and used in gene expression assays. Bead saturation studies were completed to determine the concentration of immunoprecipitating antibodies that would ensure antibody excess in immunoprecipitating the PABP-bound mRNA population (data not shown).

An issue that could complicate the precise nature of the profiling involved in mRNP-tagging systems is the potential for promiscuous exchange or reassortment of endogenous and tagged-PABP among mRNAs in cell extracts. To alleviate concern in this matter, several groups have employed the use of formaldehyde crosslinking to increase the stability of the mRNA-PABP interaction during immunoprecipitation [10–13]. However, in studies where this treatment was assessed, it was found that crosslinking had little to no effect on the level of mRNA enrichment from the target populations [9,13]. Additionally, the degree of crosslinking that is effective, without irreversibly linking the mRNA to the protein and thus dampening RNA recovery, may be difficult to determine. Published studies demonstrating that mRNA originally bound to PABP in cell lysates can not be displaced by competing excess poly(A) RNA or free PABP also alleviated concerns of PABP reassortment [9].

Nonetheless, we designed an experiment to address the degree, if any, of PABP reassortment in our system. L929 cells were pretreated with actinomycin D (AMD) for 1 h prior to infection with FLAG-PABP VRP, with infection proceeding under continued AMD treatment. AMD inhibits DNA-dependent RNA transcription, thereby preventing the synthesis of new host RNA during infection. Importantly, the treatment of cells with AMD at or shortly before alphavirus infection does not inhibit viral RNA-dependent RNA synthesis [34]. Therefore, when AMD pretreated cells were subsequently infected with FLAG-PABP VRP, the FLAG-tagged PABP was expressed from the VRP, however no new host messages were available for binding to the tagged-PABP delivered by the VRP. The only remaining source of host RNA available for binding by the VRP-supplied tagged-PABP would be host mRNA that was present prior to AMD treatment and infection—the large majority of which would already have been bound by endogenous PABP. Therefore, unless promiscuous exchange or reassortment of endogenous PABP and tagged-PABP occurred among host mRNAs, there should be little to no host RNA signal detected upon mRNA-tagging analysis of AMD-pretreated versus PBS-pretreated cells.

In fact, when the mRNA-tagging technique was used following FLAG-PABP VRP infection to isolate host mRNA from AMD-pretreated cells, either a complete absence or severely reduced levels of host mRNA in anti-FLAG immunoprecipitated lysates was observed in comparison to PBS-pretreated cells (see Figure S1). Therefore, the VRP-supplied tagged-PABP did not reassort with or out-compete endogenous PABP bound to mRNA during infection. These results, along with the data generated from other groups, indicated that PABP reassortment was not a major concern.

###  Analysis of the Message Population Isolated by VRP mRNP-Tagging

The fundamental purpose of applying an mRNP-tagging approach to the examination of the host response during viral infection is to be able to discern changes in host gene expression that occur directly within the infected cells. This is a distinct advantage over traditional profiling techniques, particularly when infected cells are the minority in the overall cell population. The mRNP-tagging technique does isolate a particular subset of mRNA in the cell, namely those that bind PABP, therefore we wanted to verify that this method would yield an mRNA population representative of host transcription following infection. To do so, cells were infected at a high MOI such that the tagging technique and the more traditional total RNA isolation would assess profiles from a similar cell population.

**Figure 3:**
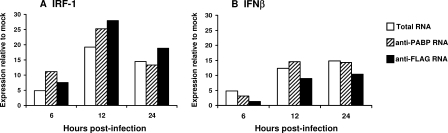
RNA Profile Comparison following High MOI Infection Changes in host gene expression were assessed following VRP infection, using RNA isolated by three distinct methods. L929 cells (10^6^) were mock treated or infected with FLAG-PABP VRP (MOI=5). At 6, 12, or 24 h, cellular RNA was harvested by three separate methods: (i) Total RNA from mock and VRP-infected cells was isolated using UltraSpec reagent. (ii) All mRNA bound to PABP in lysates prepared from mock and VRP-infected cultures were isolated by anti-PABP immunoprecipitation. (iii) mRNA from infected cells was specifically isolated by anti-FLAG immunoprecipitation, recovering FLAG-PABP bound RNA in VRP-infected lysates. The separate RNA populations served as input RNA in an RPA to analyze expression profiles of several host genes. The fold induction of (A) IRF-1 and (B) IFNβ are shown above, comparing the profiles generated from each isolation technique. For the mock references, RNA was analyzed from mock treated cells using the corresponding isolation technique (e.g., signal from infected total RNA was compared to mock total RNA, and infected IP RNA was compared to mock IP RNA). The data are a representative of two separate experiments, with each sample internally normalized to GAPDH signal.

L929 cells, a murine fibroblast cell line, were infected with FLAG-PABP VRP at an MOI of 5. At 6, 12, or 24 hours post-infection (hpi), RNA was harvested using each of the following three methods: 1) For traditional isolation of total cellular RNA, a commercially available solution of guanidine salts, urea, phenol and detergent (UltraSpec reagent) was used. 2) To isolate all poly(A) RNA bound to PABP in the cell population, an anti-PABP immunoprecipitation assay was performed on a separate set of mock and VRP-infected cells. 3) Finally, to isolate poly(A) RNA bound to the FLAG-tagged PABP provided by the FLAG-PABP VRP infection, an anti-FLAG immunoprecipitation assay was performed. RNA isolated from all three techniques was used as input RNA in an RPA designed to analyze the expression profiles of host genes relevant to this study. The infected cell profile of two such genes, *IRF-1* and *IFNβ*, relative to that in mock infected cells is shown in Figure 3, with a comparison of the levels as assayed by the three different RNA isolations. The data shown are representative of two separate experiments, and the specific mRNA signal generated from each isolation technique was normalized to *GAPDH* signal. During a timecourse of 6, 12, and 24 hpi at a high MOI, the message profiles of these two relevant host genes were similar among the various methods that were used to isolate RNA. Therefore, the mRNP-tagging technique was not limiting in terms of the availability or abundance of RNA screened.

### VRP mRNP-Tagging Provides a Sensitive Measure of Viral-Induced Host Gene Expression

**Figure 4:**
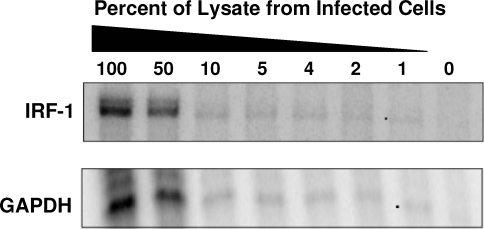
Sensitivity of the VRP mRNP-Tagging System To assess the level of sensitivity in the mRNP-tagging system, an *in vitro* experiment was performed to model the *in vivo*–like condition of a diluted infected cell population. At 6 hpi, cell lysates (1 ml) were prepared from 10^6^ L929 cells that had been infected with FLAG-PABP VRP (MOI=5). The resulting lysates were mixed in decreasing ratios of infected lysate to mock lysate, to a total volume of 200 μl. Anti-FLAG immunoprecipitation was performed to isolate RNA from the infected cell portion of this mixed lysate. To assess the mRNA signal recovered from the infected cells, an RPA was used to detect several host mRNAs, two of which are shown above (IRF-1, GAPDH). Signal from infected cell RNA within the mixed lysate was detected in samples comprised of as little as 1% infected cell lysate (highlighted by the small dots), a value approximately equal to 2 × 10^3^ infected cells. Quantitation of the IRF-1 signal can be found as supplementary data (Table S1, Figure S2).

While the RNA populations examined using the VRP mRNP-tagging system in a high MOI situation lead to informative analyses, the truly advantageous use of the technique is in low MOI situations, where the number of infected cells are a small minority (i.e., tissues from VRP-infected animals). Therefore it was important to assess the level of sensitivity that could be expected in these low MOI situations. An *in vitro* experiment was designed to model the *in vivo*–like condition of a low frequency infected cell population. At 6 hpi, cell lysates were prepared from L929 cells that were either mock infected or infected at an MOI of 5 with FLAG-PABP VRP. The cell lysates were mixed in decreasing ratios of infected cell lysate to uninfected cell lysate, and the mixed lysate was then immunoprecipitated with anti-FLAG antibody, isolating the FLAG-PABP bound mRNA. This mRNA served as template RNA in an RPA, using probes specific for several host mRNAs. The results (Figure 4) for two host mRNAs, *IRF-1* and *GAPDH*, demonstrate that the mRNP-tagging system provides a sensitive measure of VRP-induced host gene expression, as the signal from FLAG-tagged host mRNA immunoprecipitated from the infected cells was detected in samples that contained as little as 1% infected cell lysate. In this particular experiment, the 1% value is approximately equal to 2,000 infected cell equivalents. This mRNA signal can be directly visualized in Figure 4, as well as by the raw pixels plotted for IRF-1 in Table S1 and Figure S2. It is worthy to note that RPAs do not include an amplification step, and as such the input RNA is directly assayed. Therefore, a higher degree of sensitivity was expected when shifting to profiling methods that include an amplification step, such as real-time PCR.

**Figure 5:**
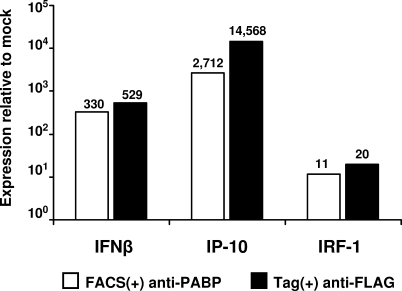
Infected Cell Gene Expression Profiles Generated by mRNP-Tagging versus FACS-Based Assays The VRP mRNP-tagging approach and a FACS-based method of sorting infected cells were compared. L929 cells (1.5 × 10^6^) were infected with either GFP-VRP or FLAG-PABP VRP (MOI=0.2). Twelve hours after GFP-VRP infection, infected cells were sorted and recovered based on GFP expression via FACS. The recovered GFP-positive (infected) cells were lysed, and all PABP-bound host messages were isolated by anti-PABP immunoprecipitation. In parallel, 12 h after FLAG-PABP VRP infection, the mRNP-tagging assay was used to directly sort the mRNA from infected cells by anti-FLAG immunoprecipitation. To evaluate and compare host gene expression in the infected cell populations, two independent samples were analyzed for IFNβ, IP-10, and IRF-1 expression by Taqman real-time PCR. The results were normalized to GAPDH signal, compared to PABP-bound mRNA from mock infected cells, and averaged.

An alternate strategy to assess host gene expression changes specifically in the infected cell compartment might rely on the sorting of the infected cell population from the uninfected cells (e.g., by fluorescence-activated cell sorting [FACS]), followed by the independent analysis of the RNA isolated from each population. To further assess the level of sensitivity, VRP-induced changes in host gene expression as assessed by the mRNP-tagging method were compared to the host profile derived by a FACS-based method (Figure 5). L929 cells (2 × 10^6^) were infected at a low MOI of 0.2 with VRP expressing either GFP (Figure 1B) or FLAG-PABP. At this MOI, it is estimated that ∼10% of L929 cells were infected (see Figure S3A). At 12 hpi, GFP expression was used as the basis for FACS-facilitated sorting of the GFP-VRP infected and uninfected cells into the two respective populations. The recovered GFP-positive (infected) cells were lysed, and all PABP-bound host messages were subsequently isolated by anti-PABP immunoprecipitation and RNA isolation. In parallel, the mRNP-tagging assay was used to sort messages from FLAG-PABP VRP infected L929 cells by lysing the entire monolayer, and using anti-FLAG immunoprecipitation to isolate FLAG-tagged PABP-bound messages specifically from the infected cells in the monolayer. To compare host mRNA levels in each infected cell population, mock treated L929 monolayers also were lysed, and the PABP-bound mRNA isolated by immunoprecipitation.

As shown by real-time PCR analysis (Figure 5), a substantial induction of *IFNβ*, *IP-10*, and *IRF-1* mRNA in the L929 infected cell population over mock treated cells was apparent following analysis of both the FACS-based or mRNP-tagging techniques. Importantly, in comparing the two methods, the degree of sensitivity in detecting mRNA from the minority population of infected cells using the tagging technique was at least equal to (*IFNβ*, *IRF-1*), if not enriched (>5× enrichment in *IP-10*) in comparison to those generated by the FACS-based method. These results further validate the mRNP-tagging system as a powerful tool for the analysis of changes in host gene expression following viral infection.

### Utilizing VRP mRNP-Tagging in Dendritic Cells Provides an *In Vitro* System for Studying Early Events in VEE Pathogenesis

A major advantage in using VRP as opposed to VEE virus in the mRNP-tagging system is the opportunity they provide to study the earliest events in the course of VEE pathogenesis, as VRP infect and replicate only within the first round of infected cells. We have previously demonstrated that dendritic cells represent an important early target of infection *in vivo*, as VRP target DCs at the site of inoculation following footpad delivery in the mouse model [20], and have likewise been shown to efficiently transduce human DCs *in vitro* [35]. In addition, several groups have examined DC-tropic properties for other alphaviruses, such as Sindbis virus [36–39] and Ross River virus [40]. Primary murine bone marrow dendritic cells were therefore chosen as an *in vitro* model system to study early VRP-induced host responses.

To compare the host response profile following traditional total RNA isolation of bone marrow–derived dendritic cells (BMDCs) versus that generated using the mRNP-tagging system, primary DCs isolated from 129sv/ev mice were infected at an MOI of 0.5 with FLAG-PABP VRP. At this MOI, approximately 4% of the cells are infected (see Figure S3B). At 6 hpi, RNA was isolated from mock treated and VRP-infected BMDC by either 1) preparing cell lysates for isolation of PABP and FLAG-PABP bound mRNAs by immunoprecipitation, or 2) adding UltraSpec reagent for isolation of total RNA. The mRNP-tagging method specifically isolated mRNA from the infected cells using the bound FLAG-tagged PABP marker, and this population of mRNA was compared to endogenously PABP-bound messages in the mock BMDC culture. In contrast, the traditional RNA isolation lacked this discrimination, and therefore total cellular RNA was isolated from the entire infected and mock treated BMDC cultures for comparison.

**Figure 6:**
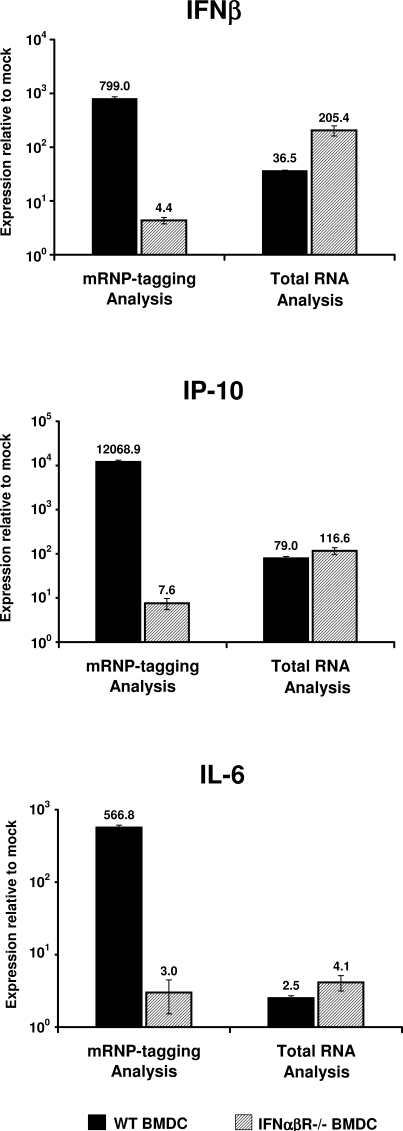
Role of Interferon Signaling in VRP-Induced Host Gene Expression in Infected versus Bystander BMDC BMDC (10^6^) generated from wildtype 129sv/ev mice (black bars) were infected with FLAG-PABP VRP at a low MOI (0.5). At 6 h, RNA was isolated from mock and VRP-infected BMDC by either 1) preparing cell lysates for isolation of FLAG-PABP-bound mRNA via anti-FLAG immunoprecipitation, or 2) using UltraSpec reagent to isolate total cellular RNA. The mRNP-tagging method specifically isolated mRNA from the minority of infected BMDC via the bound FLAG-PABP. Conversely, total RNA extraction was used to isolate cellular RNA from the entire VRP-infected BMDC culture, with the majority of the population being DCs that had not been infected. To examine the contribution of signaling through the IFNαβ receptor, the same analysis was carried out in BMDC derived from IFNαβR−/− mice (hatched bars). cDNA was generated from each RNA isolation, and assessed for changes in host gene expression by Taqman real-time PCR. Three independent samples were normalized to GAPDH signal and analyzed in comparison to mock infected BMDC: Infected cell anti-FLAG(PABP) signal was compared to mock PABP signal, and infected total RNA signal was compared to mock total RNA signal. The data are shown as the geometric mean, ± the standard error of the mean.

As shown in Figure 6 (black bars), the gene expression profiles (*IFNβ*, *IP-10*, *IL-6*) evaluated specifically from the infected cells of the BMDC culture using the mRNP-tagging technique were dramatically enhanced in comparison to profiles from the entire population of infected and uninfected DCs generated using total RNA. The fold induction of *IFNβ*, *IP-10*, and *IL-6* mRNA in the infected BMDC cultures were found to be approximately 20- to 200-fold higher than that measured by total RNA (compare the black bars in Figure 6). It is likely that the high proportion of uninfected cells in the low MOI environment masked the signal from the minority of infected cells when assayed from the total RNA samples. Therefore only once these populations could be assessed separately could the fundamental differences existing between the infected and uninfected cell responses be revealed. All three of the evaluated defense response genes were induced to high levels in the infected DCs at this early timepoint of 6 hpi, suggesting that the host innate response is rapidly initiated following VRP infection of BMDCs. Although this rapid response could be detected in the DC culture as a whole using the total RNA analysis, the mRNP-tagging method was required to reveal the full extent of this early defense response within the infected cell population.

###  In the Absence of IFNAR Signaling, the Defense Response in Infected BMDCs Is Greatly Diminished

The contribution of the IFNαβ system in determining the outcome and severity of VEE disease has been described for over 30 years [41,42]. Therefore, in seeking to further characterize the initial stages of VEE pathogenesis, the interferon response was a primary target for study. Accordingly, in the previous experiment, the host response to infection in primary BMDC isolated from IFNαβ receptor knockout (IFNαβR−/−) animals also was analyzed at 6 hpi. We hypothesized that in a system lacking IFNαβ receptor signaling, the mRNP-tagging method of specifically profiling the infected cell response should detect a diminished induction in interferon-stimulated genes (ISGs), as the positive autocrine feedback signaling within these cells should be crippled in the absence of the IFNαβ receptor. In this manner, the IFNαβR−/− BMDC also provided another model system for substantiating the VRP mRNP-tagging technique.

As demonstrated in Figure 6, the ISG response in the infected IFNαβR−/− BMDC (stippled bars) as measured by the mRNP-tagging assay was much diminished in comparison to the response in wildtype BMDC (black bars). The induction of *IFNβ* and *IP-10* mRNA in the receptor knockout BMDC was diminished by approximately 200- to 1,500–fold, respectively, in comparison to levels of induction in the wildtype BMDCs. The *IL-6* response also was reduced specifically in the infected cells of the IFNαβR−/− BMDC culture as compared to the wildtype IFNαβR+/+ BMDC culture, with the induction of *IL-6* message measured by the mRNP-tagging assay at only background levels in comparison to mock treated IFNαβR−/− BMDC culture. Therefore, the host response in the absence of IFNαβ receptor signaling did in fact demonstrate a diminished induction of ISGs, specifically within VRP-infected cells.

In contrast to the infected cell response, when the same cultures were globally assayed for changes in gene expression using total RNA analysis, the loss of the IFNαβ receptor in the culture overall appeared to have little effect on ISG induction at this early time post-infection (Figure 6). As compared to mock treated cells, the induction of *IP-10* and *IL-6* mRNA was similar in wildtype and interferon receptor knockout BMDC when measured by total RNA at 6 hpi. However, following VRP infection, *IFNβ* total RNA message levels were found to be approximately 5-fold higher in the IFNαβR−/− BMDC culture in comparison to the wildtype BMDC culture. This enhancement may be due in part to anti-viral paracrine signaling induced in the uninfected cell majority in an IFNαβ receptor-independent manner. It is important to note that in analyzing this low MOI infection using only total RNA, the high background of uninfected cells in the culture (∼94% to 96% of the culture, Figure S3B) masked the dramatic effect the receptor knockout had on host gene expression in the infected cell population. The decreased host response of *IFNβ*, *IP-10*, and *IL-6* message in the infected IFNαβR−/− cells would have gone undetected had the mRNP-tagging system not been utilized.

### *In Vivo*, the Combination of mRNP-Tagging and Traditional Profiling Reveals Dynamic Multifactorial Interactions in the DLN

Previous studies by our group and others have examined the succession of events characteristic of VEE pathogenesis in the mouse model. Following inoculation in the footpad, Langerhans-like cells infected at the site of inoculation in the footpad subsequently migrate to the popliteal DLN [20]. It has been hypothesized, based on these observations, that the early events within the DLN set the stage for the specific pattern of virus replication and host response characteristic of VEE pathogenesis. Seeking to focus on the early interactions of virus and host in the DLN, we extended our characterization of the host response to the mouse model, with the hypothesis that combining total RNA profiling with the tagging system would generate a more comprehensive view of the host response post-infection *in vivo.*

**Figure 7:**
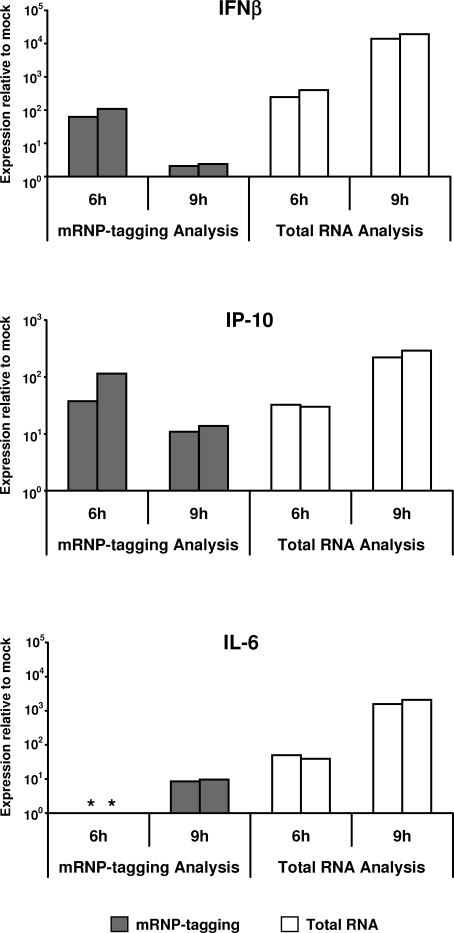
*In Vivo*, the Combination of Traditional Profiling and mRNP-Tagging Uncovers Dynamic Gene Expression Changes within the DLN Adult female Balb/c mice were inoculated in both rear footpads with 10^6^ IU of FLAG-PABP VRP. Mock treated animals were inoculated with diluent alone. At 6 and 9 h, the popliteal DLNs were removed and washed with PBS. To isolate RNA from the entire cellular population of the DLN, both DLNs were pooled, homogenized, and total RNA extracted. For isolation of mRNA specifically from the infected cells of the DLN, the mRNA tagging technique was used: Five DLNs were pooled per sample and homogenized in lysis buffer, followed by anti-FLAG immunoprecipitation. cDNA was synthesized from each RNA sample, and Taqman real-time PCR was performed against several target host genes. Two independent pools were analyzed from each group, with GAPDH serving as the internal housekeeping control gene. Infected cell anti-FLAG (PABP) signal was compared to mock PABP signal, and infected total RNA signal was compared to mock total RNA signal. The results from each independent pool are graphed side-by-side. Asterisks indicate samples from infected DLN that had no detectable signal following anti-FLAG immunoprecipitation. mRNP-tagging analysis in conjunction with traditional profiling reveals two distinct, comprehensive views of the response to infection within this target tissue *in vivo*.

Adult BALB/c mice were inoculated in each rear footpad with 10^6^ IU of FLAG-PABP-VRP. At 6 h and 9 hpi, the popliteal DLNs were removed and pooled. Subsequently, either total RNA was isolated using the RNeasy Protect protocol (Qiagen), or RNA specifically from the infected cells was isolated by anti-FLAG immunoprecipitation. cDNA was synthesized from each RNA sample, and Taqman real-time PCR was performed to analyze several target host genes. Two independent DLN samples were analyzed from each group, with *GAPDH* serving as the internal housekeeping control gene. The expression profiles of three host messages (*IFNβ*, *IP-10*, and *IL-6*) characterized from the DLN post-infection are shown in Figure 7. Two distinct views of the response to infection in the DLN were revealed; the global host response in the DLN as a whole, and the specific response within the infected cell population.

In examining the response to infection over time within the infected cells, an early robust expression of *IFNβ* and *IP-10* was exhibited in the anti-FLAG isolated RNA at 6 hpi, which waned by 9 hpi. This suggests a very rapid response to VRP infection within infected cells of the DLN. While the response was waning between 6 and 9 hpi within the infected cells, the total RNA profile of the DLN demonstrated that the response in the organ as a whole was increasing during this time interval. By 9 hpi, the majority of the *IFNβ* and *IP-10* host response appears to have shifted to the surrounding uninfected cells of the DLN. This is likely due to a robust activation of the innate immune response directly within the infected cells, which then induces mediators (e.g., cytokines) that are released into the surrounding environment of the DLN to initiate paracrine responses in neighboring uninfected cells. This detailed view of events occurring at early times post-infection has been difficult, if not impossible, to examine previously.

The *IL-6* host response presents an interesting scenario, as there was a complete lack of signal detected at 6 hpi in the infected cells of the DLN as measured by the mRNP-tagging system (Figure 7, asterisk). However there was a robust *IL-6* response measured at 6 hpi from the total RNA isolated from the entire DLN. This suggests that the infected cells of the DLN are not the main producers of *IL-6* initially following VRP infection. Instead, the uninfected bystander cells may be particularly poised to respond to paracrine signals from infected neighboring cells, resulting in *IL-6* expression. By 9 hpi, *IL-6* expression was detected in both the infected cell population as well as in the DLN as a whole, indicating a continuation of the dynamic interplay between the infected and uninfected cells in this environment. This two-phase innate response would have otherwise gone undetected had the mRNP-tagging technique not been integrated with the traditional profiling, allowing for a uniquely multifaceted examination of the VRP-infected host.

## Discussion

Here we have introduced an innovative approach for assessing gene expression changes following viral infection *in vitro* and *in vivo*, addressing a critical parameter that has been difficult, if not impossible, to address previously. By distinguishing changes in the host transcriptional program of infected cells from that of uninfected bystander cells, the mRNP-tagging technology provides an important advancement in gene expression profiling, and promises to increase our understanding of the host response to virus infection, particularly *in vivo*.

Characterization of the VRP mRNP-tagging approach has demonstrated several key aspects of the system. First and foremost is the ability of the system to effectively target and isolate mRNA from the infected cell population. In high MOI cell culture experiments, where all cells are infected, the gene expression profiles generated from total RNA and RNA isolated by mRNP-tagging were similar. Additionally, in low MOI cell culture experiments, where only a minority of cells (∼10%) are infected, the RNA profiles generated from the infected cell population isolated by FACS were similar to the gene expression profiles isolated by the mRNP-tagging technique without prior cell sorting. These results demonstrate that mRNP-tagging yields profiles that are representative of infected cells, even when they are the minority cell population.

An equally important aspect of the mRNP-tagging system is that the method is sensitive. In mixing experiments, where the infected cell signal was diluted with mock lysate, the mRNP-tagging approach detected a unique transcriptional profile when as few as 1% of the cultured cells were infected. Moreover, the sensitivity of directly isolating infected cell mRNA via the mRNP-tagging approach proved to be as great, or even enhanced in comparison to analysis of message levels when isolated from infected cells following a commonly utilized FACS-based approach. This ability to effectively analyze a small minority of infected cells, such that the mRNA signal from infected cells is no longer masked by the background of uninfected cells, is a property well suited for application *in vivo*.

Additionally, the VRP mRNP-tagging technique allows this level of specificity without harsh treatment of the infected cell populations prior to RNA isolation. This is in contrast to FACS analysis, which commonly requires physical manipulation of cultured cells or tissues (e.g., trypsinization, collagenase digestion), and may result in cellular damage to delicate cell types analyzed post-infection, such as the shearing of fragile dendrites from the cell body of DCs. This physical manipulation may affect the host gene expression profile of cells, similar to the profound effects various isolation techniques can have on the maturation and function of DCs [43]. However, since the VRP mRNP-tagging system isolates mRNA from infected cells following lysis directly in the culture vessel or from intact tissue, the cells are not distressed prior to message isolation and may more accurately reflect the host transcriptional program at the time of isolation.

Furthermore, avoiding manipulations that result in a long lag time between cells/tissue harvest and RNA isolation may be particularly advantageous when dealing with relatively unstable messages. The finding of 5-fold higher levels of IP-10 by the mRNP-tagging technique in Figure 5 may reflect such a situation. It is possible that the difference in IP-10 signal sensitivity between the GFP-VRP infected group and the FLAG-PABP VRP infected group may be a manifestation of the stability of the IP-10 message versus the other messages examined. In fact IP-10 message has been documented to be relatively unstable and to increase in stability when mRNA binding proteins are bound to it [44]. Lysing and analyzing the infected cell IP-10 message directly from the culture vessel by the mRNP-tagging method may have allowed us to increase the sensitivity in detecting an unstable message. It is also worthy to note that given the well-established roles for PABP in translation initiation and mRNA stability [5,7,8,45], PABP-associated mRNA may actually be more representative of the cell's actively translated message population or the proteome [1,13].

Application of the mRNP-tagging method has revealed several important insights into the host innate response to virus infection. First, the host response is dramatically different in infected and uninfected cells within the same *in vitro* cell culture or the same tissue *in vivo*, varying in quantitative, qualitative and temporal terms. Quantitatively, the level of response in each population varied by gene, indicating that the transcriptional programs of infected and uninfected cells within the same culture or tissue are uniquely affected. On a gene by gene basis, comparing the infected cell profile generated by the mRNP-tagging system to the total RNA profile generated from the entire culture or tissue allows the relative contribution of the infected versus uninfected cell populations to be teased apart. In the case where a small percentage of primary BMDC were infected *in vitro*, the induction of *IFNβ*, *IP-10*, and *IL-6* in infected cells as measured by the mRNP-tagging system was up to 200 times that indicated in the total RNA sample for the entire culture. These results suggest that the infected cells may be the primary source of the early IFNβ, IP-10, and IL-6 response to VRP infection in BMDC cultures.

Additionally, had the mRNP-tagging system not been utilized, the induction of *IL-6* would likely have been overlooked due to the low total RNA induction measured from the BMDC culture. Therefore, at particular times the level of response in the infected and uninfected cell populations can differ so extensively that their analysis becomes qualitatively different. In other words, a response that is robust in one population may be completely absent in the other, and in this regard, the host response will appear to be quite different when evaluating infected cells alone rather than the entire culture or tissue.

Temporally, the combination of the mRNP-tagging approach and total RNA analysis offered a unique vantage point into the kinetics of the infected and uninfected cell responses. In the DLN, this analysis demonstrated two phases of the innate host response. The first apparent phase was a rapid response in the infected cells of the DLN, including the robust activation of key host defense genes, *IFNβ* and *IP-10*, at 6 h following footpad inoculation. While this infected cell response was waning by 9 hpi, an apparent second phase of response was mounting in the surrounding uninfected cells of the DLN, with induction of the same defense genes increasing from 6 h to 9 hpi. In fact, a similar rapid onset of the host innate response has been described previously following virulent VEE infection, with cytokine RNA levels in the DLN of infected mice peaking at 6 to 12 h following footpad inoculation, and waning by 24 to 48 h [46]. However, the use of VRP and the application of the mRNP-tagging system in our study provided the ability to distinguish the events specifically occurring within the first round of infected cells *in vivo* from the effects on the uninfected bystander cells of the DLN, as well as from the complication of viral cell-to-cell spread, revealing the unique kinetic response to infection occurring in each population.

Several signaling events are likely to contribute to the kinetics of this two-phase activation. Replicon particle binding, entry, and replication provide a multitude of signals to initiate the early host defense response directly within the infected cells. It is likely that the rapid innate activation of the infected cell population leads to the secretion of cytokines and other soluble immune modulators. A portion of these mediators likely initiate the cell signaling events in uninfected bystander cells that are responsible for the strong paracrine response in the DLN at later times post-infection. This would include the activation of cells which have homed to the DLN as an active site of viral infection. In fact, a large influx of cells to the DLN has been observed following footpad VRP inoculation, including cells of the proinflammatory response and antigen presenting cells (J. M. Thompson, A. C. Whitmore, J. L. Konopka, T. P. Moran, and R. E. Johnston, unpublished data). These recruited cells would remain uninfected, but would be susceptible to the primed environment of the DLN, and likely contribute to the induction of the second phase of the host innate response *in vivo*.

Our results highlight the role of the innate immune response during VEE infection, particularly the interferon response. Evidence for the major role of the interferon system in controlling VEE replication and spread *in vivo* has been well established [18,19,23,40–42,47–49], with tremendous levels of soluble, biologically active interferon in the serum, measured at up to 80,000 IU/ml following virulent VEE infection [18]. The absence of interferon signaling *in vivo* results in a significantly shorter average survival time following VEE infection (30 h in mice lacking the IFNαβ receptor in comparison to 7.7 days in wildtype mice), and a 10,000-fold increase in virus titers [18]. Here, the role of the interferon response in autocrine and paracrine signaling of infected and uninfected cells was further elucidated in BMDC. While ablating the IFNαβ receptor had no apparent effect on the total RNA induction of *IFNβ*, *IP-10*, and *IL-6* genes in the BMDC culture as a whole, a dramatically reduced induction of each of these host response genes was observed in the infected BMDC population. This strongly suggests that autocrine signaling through the IFNαβ receptor on the infected cells plays a critical role in inducing a strong ISG response.

Conversely, in the uninfected BMDC population, the absence of IFNαβ receptor signaling had little effect on the induction of the same host response genes, remaining at near wildtype levels. This was a surprising, nonetheless interesting result that suggests interferon-mediated signaling through the IFNαβ receptor is not the primary paracrine mediator leading to the induction of these particular host response genes in uninfected bystander cells. It is important to highlight the fact that this data does represent a temporal snapshot of the host response at the early time of 6 h post VRP infection. However, it is consistent with previous findings showing levels of biologically active type 1 serum interferon are similar in IFNαβ receptor knockout and wildtype mice at early times post VRP infection [18]. Together, these data suggest that at least early in infection, VRP may induce an interferon response mediator that is independent of type 1 receptor signaling, such that surrounding uninfected cells lacking the type 1 receptor are still capable of mounting an antiviral response. It is important to note that BMDC lacking the type 1 interferon receptor are more permissive to infection with VRP than wildtype BMDC (Figure S3B), which may result in increased levels of transgene expression (Figure S3C), and may in part contribute to the higher induction of *IFNβ* in the receptor knockout BMDC demonstrated in Figure 6. Such factors will need to be addressed should a separate line of investigation be initiated to explore the identity of this novel mediator(s).

Taken together, the data presented here highlight an important additional observation. Namely, reductionist *in vitro* approaches do not always recapitulate what is occurring *in vivo*. Often, high multiplicity infections of largely homogenous cultured cells are utilized *in vitro* to draw conclusions about what occurs in naturally low multiplicity infections of complex heterogeneous tissues *in vivo*. However, the data presented here strongly argue that the naturally heterogeneous environment of infected and uninfected cells existing during infection *in vivo* must be appreciated in order to understand the dynamic interactions occurring between these populations. For example, here in cultured BMDC, *IL-6* was highly induced in VRP infected cells at 6 hpi. However, this did not appear to be the case *in vivo* where induction of *IL-6* mRNA was first documented in the uninfected bystander population.

The application of the VRP mRNP-tagging system offers a multitude of future studies that promise unique perspective on the highly coordinated host response to viral infection. However, it is worthwhile to note that there is room for potential improvement to the overall VRP mRNP-tagging platform. As it stands currently, the mRNP-tagging system specifically grants a temporal snapshot of the infected cell response—a response that differs not only quantitatively but also qualitatively from that of the bystander uninfected cells and from the culture as a whole. While this is a key strength, in this initial application of the system we relied on total RNA isolated by a separate technique to evaluate the “uninfected cell” response. This was possible given an underlying condition of low MOI infections, in that the majority of cells in a given culture or tissue would be uninfected. In evolving the system from here, a major goal will be to achieve a way to more directly compare host RNA responses from uninfected and infected cells in the same culture or tissue. Nonetheless, even as it stands at this current stage, the mRNP-tagging system provides a powerful way to address the dynamic interaction of pathogen and host.

A critical future application of the VRP mRNP-tagging system will be the analysis of the host response to infection within the brain, where the most extensive pathogenesis is observed following VEE virus infection. While alphavirus CNS pathogenesis has been studied extensively in several model systems, it has been suggested that multiple host- and virus-specific parameters contribute to VEE-induced pathology in the brain [50–52]. In elucidating such parameters, distinguishing the relative contribution of infected versus bystander cells in the CNS has proven critical following infection with neuroadapted Sindbis virus [53,54], and is also likely to be crucial in understanding VEE-induced neurodegeneration. Developing the mRNP-tagging system using VEE virus with a double 26S subgenomic promoter would also facilitate the characterization of the host response to VEE infection downstream of the DLN, including the impacts of virus budding as well as cell-to-cell spread during infection [20].

The application of mRNP-tagging technology to the study of the host response to viral infection opens avenues of investigation that have previously been difficult to navigate. A better understanding of virus–host interactions may subsequently facilitate the design of improved therapeutics and vaccines. More specifically, gaining a clear profile of the host response to VEE infection promises to further our understanding of the specific virus–host interactions that define alphavirus pathogenesis *in vivo*.

## Materials and Methods

### VEE replicon particles (VRP).

The construction and packaging of VRP using a split helper system have previously been described [33]. The replicon plasmid constructs used in this study were (i) replicons expressing green fluorescent protein (GFP-VRP) and (ii) replicons expressing an N-terminally FLAG-tagged version of poly(A) binding protein I (FLAG-PABP VRP). The production of GFP-VRP has been described previously [20,55]. The FLAG-PABP replicon plasmid was generated by the directional cloning of the ORF of *PABP I* containing an N-terminal FLAG epitope tag (GACTACAAGGACCACGATGACAAG, kindly provided by J. D. Keene [14]), immediately downstream of the 26S mRNA promoter of the pVR21 replicon plasmid.

All replicon particles used in this study were packaged in the wildtype (V3000) VEE envelope [33]. Briefly, the replicon RNA genome containing the VEE nonstructural genes and expressing the heterologous gene from the viral 26S promoter, along with two defective helper RNAs providing the wildtype capsid and glycoprotein genes, but lacking the virus-specific packaging signal, were co-electroporated into BHK-21 cells (ATCC). Due to the lack of encoded viral structural genes in the replicon genome, infectious VRP undergo only one round of infection, and the absence of propagating recombinant virus was confirmed by passage in BHK-21 cells. VRP were concentrated from supernatants by centrifugation through a 20% sucrose cushion and resuspended in PBS. BHK-21 titers were determined either by immunofluorescence (GFP-VRP), or immunocytochemistry (null VRP, FLAG-PABP-VRP) using sera containing antibody to the VEE nonstructural proteins.

### Cells and in vitro infections.

*(i) Infection of L929 cells.* L929 murine fibroblasts (ATCC) were maintained at 37°C under 5% CO_2_ in complete alpha minimal essential medium (αMEM, Gibco) containing 10% donor calf serum, 10% tryptose phosphate broth, 2 mM L-glutamine, 100 U/ml penicillin and 0.5 mg/ml streptomycin. For VRP infection, 10^6^ cells were seeded in 60 mm dishes and incubated overnight. The medium was removed from the monolayer and the cells were infected at a multiplicity of infection (MOI) of 5 (unless otherwise indicated) in 0.2 ml endotoxin-free PBS supplemented with 110 mM Ca^2+^, 50 mM Mg^2+^, and 1% vol./vol. donor calf serum. After 1 h of adsorption at 37°C, complete αMEM was added to the monolayer. In studies involving the pretreatment of L929 cells with AMD to inhibit the transcription of new host RNA, cells were pretreated with 4 ug/ml AMD for 1 h prior to VRP infection.

*(ii) Generation of primary murine BMDCs.* Breeding pairs of IFNαβR+/+ 129Sv/Ev and IFNαβR−/− mice were kindly provided by Herbert Virgin (Washington University, St. Louis, MO) and Barbara Sherry (North Carolina State University, Raleigh, NC), respectively. Mice were bred under specific pathogen-free conditions in the Department of Laboratory Animal Medicine breeding colony facilities at the University of North Carolina, Chapel Hill. To generate primary immature BMDCs [40,56], bone marrow cells from femurs and tibia of 8- to 14-week-old mice were aspirated with RPMI-10 medium (RPMI 1640 [Gibco], 10% FBS, 2 mM L-glutamine, 50 uM 2-ME, 100 U/ml penicillin, 100 ug/ml streptomycin sulfate). Cells were filtered through a 40 um cell strainer, pelleted, and resuspended in lysis buffer (0.15 M NH_4_Cl, 0.1 mM Na_2_EDTA, 1 mM KHCO_3_ [pH 7.2–7.4]). Following lysis of red blood cells at room temp, 10 ml of RPMI-10 media/mouse was added, cells were again pelleted and resuspended in fresh RPMI-10 media. Cells were seeded in 6-well low cluster plates (Corning) in RPMI-10 media supplemented with 20 ng/ml GM-CSF (Peprotech), and incubated at 37°C under 5% CO_2_. On day three, RPMI-10 media supplemented with 20 ng/ml GM-CSF and 20 ng/ml IL-4 (Peprotech) was added to each well. On day five, additional RPMI-10 media supplemented with 10 ng/ml GM-CSF and 10 ng/ml IL-4 was added to each well. On day seven, the BMDC were harvested and either immediately used for infection, or were cryopreserved at 2–5 × 10^6^/ml in 90% FBS/10% DMSO.

*(iii) Infection of primary BMDCs.* Cryopreserved BMDCs were quickly thawed in a 37°C water bath, and gently transferred to a conical tube containing an equal volume of RPMI-10. The volume of RPMI-10 was brought up to 10 ml, and the cells were pelleted. An additional wash with RPMI-10 was completed, and the cells were resuspended in RPMI-10, supplemented with 5 ng/ml GM-CSF and IL-4. BMDCs were seeded at 2.5 × 10^6^ cells/well in hydrated six-well low cluster plates, and allowed to recover overnight at 37°C, 5% CO_2_. BMDCs were harvested and pooled with a cold PBS wash of each well. After pelleting, cells were resuspended at 10^6^ cells/ml, and 10^6^ cells/well were seeded in a hydrated six-well low cluster plate. BMDC were infected with VRP at an MOI of 0.5 in 100 ul PBS supplemented with 1% donor calf serum and Ca^+^/Mg^+^. Following 2 h of absorption at 37°C, 5 ml of RPMI-10 supplemented with 5 ng/ml GM-CSF and IL-4 was added to each well.

### Animals and in vivo infections.

Seven- to eight-week-old female BALB/c mice were obtained commercially (Charles River Laboratories) and allowed to acclimate for 5–7 days. Mice were inoculated in each rear footpad with 10^6^ IU of VRP diluted in 10 ul endotoxin-free PBS containing 1% donor calf serum. Mock-infected animals received diluent alone.

### Total RNA isolation.

*(i) L929 cells.* At indicated times post-infection, media was removed and L929 cell monolayers were washed with cold PBS. The UltraSpec RNA Isolation System was used to isolate total RNA, with 1ml of UltraSpec RNA Reagent added to each 60mm dish of L929 cells per manufacturer's protocol (Biotecx).

*(ii) Primary BMDCs.* At 6 or 12 hpi, BMDCs were transferred to a 15 ml conical tube and pelleted (1,200 rpm, 10 min at 4°C), during which time each well was washed with cold PBS. The wash was used to resuspend the pelleted cells, followed by a second spin. Total RNA was harvested from BMDC using the RNeasy Mini Kit, according to the manufacturer's protocol (Qiagen).

*(iii) Lymph nodes.* At indicated times post infection, mice were euthanized and both draining popliteal lymph nodes were harvested, washed with cold PBS, and pooled together into 200 ul RNA*later* RNA Stabilization Reagent (Qiagen, Ambion). Total RNA was harvested from tissue homogenate prepared using a plastic pestle with a handheld motor and the RNeasy Protect Mini Kit (Qiagen).

### mRNA-tag immunoprecipitation and RNA isolation.

*(i) Antibodies.* The mRNP-tagging method as applied to cells and animal tissues infected with VRP was developed from general ribonomics/mRNP-tagging protocols previously described by the Keene laboratory [1,2,9,14]. Polyclonal anti-PABP antibody was generously provided by J. Keene (Duke University Medical Center). Additionally, polyclonal anti-PABP H-300 antibody was obtained from Santa Cruz Biotechnology. Monoclonal anti-FLAG M2 antibody was acquired from Sigma-Aldrich.

*(ii) Preparation of mRNP lysate from cultured cells.* L929 monolayers (10^6^ cells total) were washed with cold PBS, followed by lysis of the monolayer with 1ml of polysome lysis buffer (100 mM KCl, 5 mM MgCl2, 10 mM Hepes [pH 7.0], and 0.5% Nonidet P-40 with 1 mM DTT, 100 U/ml RNaseOUT [Invitrogen], 0.2% vanadyl ribonucleoside complex [New England Biolabs], and 1 tablet/10 ml Complete Mini Protease Inhibitor Cocktail Tablet [Roche] added fresh at time of use). For BMDC lysis, cells were gently pelleted and the media removed. The pellet was washed with PBS and spun again, followed by resuspension and lysis in 500 ul of polysome lysis buffer. Cells were lysed for 10 min, followed by centrifugation at 14,000*g* in a tabletop microfuge for 10 min at 4°C to remove cellular debris. The ∼1 ml total volume of L929 mRNP lysate (isolated from 10^6^ L929 cells) was stored at −80°C in 200 ul working aliquots, while the 500 ul total volume of BMDC mRNP lysate (isolated from 10^6^ BMDC cells) was stored at −80°C in 250 ul working aliquots.

*(iii) Preparation of mRNP lysate from whole animal tissue (DLN).* Freshly dissected DLN were washed with ice-cold PBS. Five DLN were pooled per sample, and coarsely homogenized in 200 ul polysome lysis buffer (containing RNase and protease inhibitors as described above) using a plastic pestle and hand-held motor. Samples were frozen at −80°C until use. Upon thawing (on ice), the homogenization and lysis was continued to completion (as monitored microscopically), and the lysate spun at 4°C to pellet any remaining tissue/debris. The supernatant was transferred to a fresh tube on ice, and a second round of lysis/ homogenization was completed on the pellet using 100 ul polysome lysis buffer. Upon centrifugation, this supernatant was pooled with the first (∼300 ul total) and kept on ice until use.

*(iv) mRNP immunoprecipitation.* For each immunoprecipitation sample, two 60 ul aliquots of protein beads were prepared; the first aliquot to coat with the specified antibody, and the second to pre-absorb the lysate for removal of non-specific binding. For antibody coating, 60ul Protein-A Sepharose beads (pre-swollen with 12ml PBS/1.5g beads, Sigma) or fast flow Protein-G Sepharose beads (commercially pre-swollen, Sigma) were washed with PBS (10 volumes), followed by a second wash of 10 volumes NT2 buffer (50 mM Tris [pH 7.4], 150 mM NaCl, 1 mM MgCl2, 0.05% Nonidet P-40) tumbled end-over-end for 15 min at room temperature. Beads were resuspended in 10 volumes fresh NT2 buffer supplemented with 5% BSA, and tumbled overnight at 4°C with excess immunoprecipitating antibody (200 ul anti-PABP antibody; 25 ul anti-FLAG antibody). Prior to the immunoprecipitation reaction, the antibody-coated beads were washed with 1 ml NT2 buffer.

To pre-absorb the lysate, a second 60 ul aliquot of beads (per sample) was prepared as described above through the 15 min NT2 buffer wash. These beads were resuspended in 5–10 volumes NT2 buffer supplemented with 100 U/ml RNaseOUT, 0.2% vanadyl ribonucleoside complex, 2 mM DTT, and 25 mM EDTA. The mRNP lysate was added (∼200 ul L929 lysate, 250 ul BMDC lysate, or 300 ul DLN lysate), along with 1 ul normal animal serum corresponding to the immunoprecipitating antibody (normal mouse serum for anti-FLAG immunoprecipitation samples; normal rabbit serum for anti-PABP immunoprecipitation samples). Samples were tumbled at room temperature for 1 h to remove any material that would non-specifically bind to the beads. This pre-absorbed lysate was recovered by gently pelleting the beads and applying the supernatant to the specific antibody-coated beads, along with 5–10 volumes of NT2 buffer supplemented as described above. This immunoprecipitation slurry was tumbled end-over-end at room temperature for 2 h.

*(v) RNA extraction from immunoprecipitated mRNP complex.* Immunoprecipitated samples were gently centrifuged to pellet the beads, and washed four times with ice-cold NT2 buffer (10 bead volumes). Washed beads were resuspended in 600 ul proteinase K digestion buffer (100 mM Tris [pH 7.5], 12.5 mM EDTA, 50 mM NaCl, 1% SDS), plus 25 ul of 20 mg/ml proteinase K, and incubated for 30 min in a rotating device at 50°C. 600 ul phenol-chloroform-isoamyl alcohol (Fisher) was added to the beads, which were vortexed for 2 min and then centrifuged for 5 min at 14,000*g*, 4°C. This was followed by extraction with one volume of RNA-grade chloroform (Fisher), and standard isopropanol precipitation including 8 ul of glycogen. The samples were stored at −80°C until use for gene expression analysis. Upon thawing on ice, the samples were spun for 30 min (14,000*g*, 4°C), and the RNA pellet washed with 100 ul of 80% ethanol. The RNA pellet was resuspended in RNase-free water (Ambion), or hybridization buffer as necessary.

### RNase protection assay (RPA).

RNase protection assays were used to determine the relative abundance of specific cellular mRNAs in infected and mock infected L929 cells. ^32^P-labeled RNA probes were synthesized by use of the RiboQuant *in vitro* transcription kit and a RiboQuant multiprobe custom template set (BD Pharmingen). This custom set included template for the synthesis of radiolabeled probes specific to murine interferon beta (*IFNβ*) and interferon regulatory gene-1 (*IRF-1*), as well as mRNAs encoding the murine housekeeping proteins *GAPDH* and *L32*. RNA isolated from the mRNP complexes was used as input RNA for the mRNP-tagging samples, using 100% of the isolated RNA by resuspending the RNA pellet directly in hybridization solution. 2 ug of total RNA to be used as input samples for total RNA analysis was isolated as described above. The custom probe set was mixed with each RNA sample, placed in a pre-warmed heat block at 90°C which was immediately turned down to 56°C, and incubated overnight. The RNA-probe mixtures were treated with RNase according to the RiboQuant RPA kit protocol (BD Pharmingen). The protected dsRNA species were electrophoresed on a 4.5% polyacrylamide-8M urea sequencing-sized gel, the gels were dried, and analysis conducted on a Molecular Dynamics Storm phosphorimager with ImageQuant software. Values represent the fold change over mock expression, as normalized to anti-PABP immunoprecipitated *GAPDH* housekeeping mRNA levels for the mRNP-tagging samples, or total *GAPDH* housekeeping RNA levels for total RNA samples.

### Western blotting.

NP-40 protein lysate preparations were separated by 8% SDS-PAGE, and transferred to polyvinylidene difluoride membrane at 10 V for 60 min in transfer buffer (48 mM Tris, 39 mM glycine, 10% methanol). The membranes were blocked overnight in 5% dry milk in TBST (50 mM Tris-HCl [pH 7.4], 150 mM NaCl, and 0.1% Tween 20), followed by incubation for 1h with either anti-PABP (Santa Cruz Biotechnology, sc-28834) or anti-FLAG (Sigma-Aldrich, F3165) primary antibody diluted in 1% dry milk-TBST. The appropriate horseradish peroxidase (HRP)-conjugated secondary antibodies (Amersham Pharmacia) were then added and incubated for 1 h, followed by chemiluminescence detection using ECL detection reagents (Amersham Pharmacia). Blots were exposed to film and developed.

### Flow cytometry analysis.

For quantification of VRP infectivity and 26S transgene expression levels, GFP-VRP or mock infected cells (BHK, L929, or BMDC) were harvested at 12 hpi, and washed once with cold PBS. The cells were fixed with PBS/1% paraformaldehyde before FACS analysis. FACS data were acquired using a Dako CyAn Flow Cytometer and analyzed using Summit software (Dako).

To compare the infected cell host message profile generated by the mRNP-tagging approach versus FACS-facilitated sorting, 1.5 × 10^6^ L929 cells were mock treated or infected at a low MOI of 0.2 with either FLAG-PABP VRP or GFP-VRP. At 12 hpi, cultures were treated in one of two ways to generate a profile of host message levels specifically from the infected cells. The cell monolayers that had been infected with FLAG-PABP VRP were lysed, and FLAG-tagged host messages were immunoprecipitated from the infected cells per the mRNP-tagging protocol described above using 100% of the monolayer lysate. A separate anti-PABP immunoprecipitation was completed from both mock- and FLAG-PABP VRP-infected cell lysates to isolate all PABP-associated mRNA and served as the mock reference with normalization to *GAPDH* signal levels.

To generate a comparative profile of the infected cell host response, cell monolayers that had been infected with GFP-PABP VRP were prepared for FACS-based analysis. The monolayers were trypsinized, washed with media (αMEM supplemented with 2% FBS, 100 U/ml penicillin, and 0.5 mg/ml streptomycin) and resuspended in 700 ul of fresh media. The UNC-CH Flow Cytometry Core Facility provided cell sorting capability using the Dako Modular Flow (MoFlo) Cell Sorter, gating on GFP-positive signal to sort and recover the infected cell population. This GFP-positive cell population was gently pelleted and resuspended in 0.5 ml polysome lysis buffer. 100% of the resulting lysate was immunoprecipitated with anti-PABP antibody to isolate all PABP-associated messages specifically from the (GFP+ sorted) infected cell population. A separate anti-PABP immunoprecipitation was completed from mock cell lysate and served as the mock reference with normalization to *GAPDH* signal levels. The host message populations isolated from each technique were analyzed using Taqman real-time PCR (see below).

### cDNA synthesis, real-time PCR, and analysis.

*(i) cDNA synthesis.* A one-tube DNase treatment and reverse transcription protocol was used to generate cDNA, using SuperScript III Reverse Transcriptase First Strand cDNA kit (Invitrogen). 0.5–0.75 ug of either mRNA-tag isolated or total RNA served as input RNA for the reaction, using RNase-free water to bring the total volume to 10 ul. This was combined with 1 ul 10 mM dNTP mix (Amersham Biosciences), 4 ul 5X SuperScript III reverse transcriptase buffer, 1 ul 0.1 mM dTT, 1 ul 40 U/ul RNaseOUT (Invitrogen), and 1 ul RQ1 RNase-free DNase (Promega). The samples were DNase treated at 37°C for 30 min, followed by the addition of 1 ul RQ1 Stop Solution (Promega) and heat inactivation of the samples at 65°C for 10 min. Following the addition of random hexamer primers (150 ng, Invitrogen), reverse transcription of the samples was continued in the same tube, according to the SuperScript III protocol (Invitrogen). cDNA samples were stored at −20°C.

*(ii) Real-time PCR.* Real-time PCR was performed to determine the relative abundance of specific cellular mRNAs in infected and mock treated samples. Taqman Gene Expression Assays (Applied Biosystems) containing primers and probes for various target host messages were used, with each reaction performed in a 25 ul total volume (5 ul cDNA, 12.5 ul TaqMan Universal PCR Master Mix without AmpErase UNG [Applied Biosystems], 1.25 ul probe/primer mix [Applied Biosystems], and 6.25 ul RNase-free water). The default amplification profile was performed by the ABI Prism 7000 Real-Time PCR System, and the data converted into cycle threshold (C_T_) values by the 7000 Sequence Detection Software (v1.2.3, Applied Biosystems). Duplicate samples were amplified from each experimental group with *GAPDH* serving as the housekeeping control along with each target gene of interest. A negative template control also was performed, with all samples run in parallel on the same plate.

*(iii) Total RNA real-time PCR analysis.* Real-time PCR results are presented as fold gene expression in the infected sample over that in the mock sample, as normalized to the *GAPDH* housekeeping gene. During each reaction, a cycle threshold (C_T_) value was generated for the target gene of interest (and *GAPDH*), corresponding to the cycle number at which the fluorescence of the PCR product reached significant levels above the background threshold level. Raw C_T_ values generated from total RNA samples were analyzed using the well established delta C_T_ (ΔC_T_) method to generate the fold expression results (*User Bulletin*, ABI Prism 7000 Sequence Detection System [Applied Biosystems]). Briefly, for each cDNA sample, the GAPDH C_T_ value was subtracted from the C_T_ value of the target gene (e.g., cytokine) of interest, yielding a ΔC_T_ value. The ΔC_T_ value generated for the VRP-infected sample was then subtracted from the ΔC_T_ value of the mock sample, yielding a ΔΔC_T_ value. This widely-used method assumes the target and housekeeping genes were amplified with the same efficiency, thus the increase in host mRNA levels in the infected samples compared to the mock treated samples was calculated as 2^−(ΔΔCT )^.

*(iv) Real-time PCR analysis of mRNA-tagging samples.* Prior to this standard ΔC_T_ analysis, raw C_T_ values generated from mRNA-tagging samples were normalized in a manner that was inherently required for this system. The mock signal values were generated from an anti-PABP immunoprecipitation which isolates all PABP-bound messages in the entire cell culture or tissue analyzed. However, the infected sample values were derived using an anti-FLAG immunoprecipitation to specifically isolate the infected-cell minority subset of the population. Therefore, an mRNP-tagging normalization step was utilized to account for two parameters: 1) The disparity in the cell population size of the mock and infected samples assayed by the mRNP-tagging system, and 2) any difference in the immunoprecipitating antibody strength (the polyclonal anti-PABP antibody versus the monoclonal anti-FLAG antibody). To do so, raw *GAPDH* C_T_ values were generated from mRNP-tagging samples in the following manner. From FLAG-PABP VRP infected samples, raw *GAPDH* C_T_ values were generated from both anti-FLAG and anti-PABP immunoprecipitation-derived cDNA, representing *GAPDH* expression in the infected cell subset or the entire culture, respectively. However from mock samples, raw *GAPDH* C_T_ values were solely generated from anti-PABP immunoprecipitation-derived cDNA, thus representing the expression of *GAPDH* in the entire cell population.

Therefore, to normalize the mock control values for comparison to the infected samples, a ratio of the anti-FLAG *GAPDH* signal to the anti-PABP *GAPDH* signal was applied to account for the difference in cell population size and antibody strength (*r*):

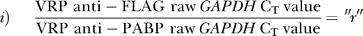
This ratio was then applied to the mock anti-PABP raw *GAPDH* C_T_ value to generate a normalized mock *GAPDH* value:

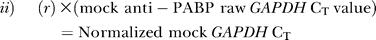



This normalized C_T_ value served as the input mock *GAPDH* value for the standard ΔC_T_ analysis, as described above for total RNA.

## Supporting Information

Figure S1 FLAG-PABP Does Not Reassort with Endogenous PABP To address the degree, if any, of PABP reassortment in our system, L929 cells were pretreated with AMD (4 ug/ml) for 1 h prior to infection with FLAG-PABP VRP. AMD inhibits DNA-dependent RNA transcription, thereby preventing the synthesis of new host RNA during infection, but does not inhibit viral RNA-dependent RNA synthesis. AMD-pretreated cells were subsequently infected with FLAG-PABP VRP, and anti-FLAG immunoprecipitation was used to isolate host mRNA from either PBS or AMD-pretreated infected cells at 2 and 5 h following FLAG-PABP VRP infection. The expression level of various host messages were analyzed by RPA, and the raw pixel values quantified for IRF-1 mRNA expression is presented above as representative data. Under AMD treatment, a complete absence of host mRNA in anti-FLAG immunoprecipitated lysates was observed in comparison to PBS-pretreated cells. Therefore, the VRP-supplied tagged-PABP did not reassort with endogenous PABP for binding to host mRNA.
(13 KB PDF)

Figure S2 mRNP-Tagging Sensitivity: IRF-1 Quantitation (Figure 4) Table S1 and Figure S2 represent quantitation of the IRF-1 signal from the RPA gel depicted in Figure 4. The raw pixel values are listed, as well as plotted against the corresponding percentage of the lysate assayed that was from infected cells, to allow further assessment of the level of sensitivity in the mRNP-tagging system. Experimental details: At 6 hpi, cell lysates (1 ml) were prepared from 10^6^ L929 cells that had been infected with FLAG-PABP VRP (MOI=5). The resulting lysates were mixed in decreasing ratios of infected lysate to mock lysate, to a total volume of 200 μl. Anti-FLAG immunoprecipitation was performed to isolate RNA from the infected cell portion of this mixed lysate. To assess the mRNA signal recovered from the infected cells, an RPA was used to detect the expression levels of host mRNA. Signal from infected cell RNA within the mixed lysate was detected in samples comprised of as little as 1% infected cell lysate, a value approximately equal to 2 × 10^3^ infected cells. Band signal quantitation was completed using ImageQuant software (GE Healthcare).
(6 KB PDF)

Figure S3 Comparative VRP Infectivity Data for BHK, L929, and BMDC at High and Low MOI To compare the level of permissiveness of different cell types for infection by VRP, flow cytometry analysis was completed using GFP-VRP at high and low MOI. 10^5^ BHK, L929, or BMDC were either mock infected or infected with GFP-VRP, and harvested at 12 hpi. The cells were washed once with cold PBS, and fixed with PBS/1% paraformaldehyde before FACS analysis. FACS data were acquired using a Dako CyAn Flow Cytometer, with GFP serving as the marker for infected cells. The data were analyzed using Summit software (Dako), and are represented as the geometric mean (n=3) ± the standard error of the mean. (A) BHK and L929 infectivity data. (B) Relative infectivity of wildtype versus IFNαβR−/− primary BMDC. (C) Relative mean fluorescence intensity of GFP expressed from the 26S promoter of wildtype versus IFNαβR−/− primary BMDC.
(12 KB PDF)

Table S1 IRF-1 Signal Quantitation (Figure 4) (13 KB XLS)

### Accession Numbers

The NCBI GenBank (http://www.ncbi.nlm.nih.gov/Genbank/index.html) accession and GeneID numbers for genes mentioned in the text are *GAPDH* (NM_008084.2, GeneID 14433); *IFNβ* (NM_010510.1, GeneID 15977); *IL-6* (NM_031168.1, GeneID 16193); *IP-10* (NM_021274.1, GeneID 15945); *IRF-1* (NM_008390.1, GeneID 16362).

## Abbreviations

BMDC: bone marrow–derived dendritic cell
CT: cycle threshold
DLN: draining lymph node
FACS: fluorescence-activated cell sorting
GFP: green fluorescent protein
hpi: hours post-infection
IFN: interferon
ISG: interferon-stimulated gene
MOI: multiplicity of infection
PABP: poly(A) binding protein
RPA: RNase protection assay
RNP: ribonucleoprotein, RNA binding protein
VEE: Venezuelan equine encephalitis virus
VRP: VEE replicon particles

## References

[ppat-0030199-b001] Tenenbaum SA, Carson CC, Lager PJ, Keene JD (2000). Identifying mRNA subsets in messenger ribonucleoprotein complexes by using cDNA arrays. Proc Natl Acad Sci U S A.

[ppat-0030199-b002] Tenenbaum SA, Lager PJ, Carson CC, Keene JD (2002). Ribonomics: identifying mRNA subsets in mRNP complexes using antibodies to RNA-binding proteins and genomic arrays. Methods.

[ppat-0030199-b003] Deo RC, Bonanno JB, Sonenberg N, Burley SK (1999). Recognition of polyadenylate RNA by the poly(A)-binding protein. Cell.

[ppat-0030199-b004] Bernstein P, Ross J (1989). Poly(A), poly(A) binding protein and the regulation of mRNA stability. Trends Biochem Sci.

[ppat-0030199-b005] Burd CG, Dreyfuss G (1994). Conserved structures and diversity of functions of RNA-binding proteins. Science.

[ppat-0030199-b006] Gorlach M, Burd CG, Dreyfuss G (1994). The mRNA Poly(A)-binding protein: localization, abundance, and RNA-binding specificity. Exp Cell Res.

[ppat-0030199-b007] Sonenberg N, Dever TE (2003). Eukaryotic translation initiation factors and regulators. Curr Opin Struct Biol.

[ppat-0030199-b008] Imataka H, Gradi A, Sonenberg N (1998). A newly identified N-terminal amino acid sequence of human eIF4G binds poly(A)-binding protein and functions in poly(A)-dependent translation. Embo J.

[ppat-0030199-b009] Penalva LO, Tenenbaum SA, Keene JD (2004). Gene expression analysis of messenger RNP complexes. Methods Mol Biol.

[ppat-0030199-b010] Roy PJ, Stuart JM, Lund J, Kim SK (2002). Chromosomal clustering of muscle-expressed genes in Caenorhabditis elegans. Nature.

[ppat-0030199-b011] Kunitomo H, Uesugi H, Kohara Y, Iino Y (2005). Identification of ciliated sensory neuron-expressed genes in Caenorhabditis elegans using targeted pull-down of poly(A) tails. Genome Biol.

[ppat-0030199-b012] Pauli F, Liu Y, Kim YA, Chen P-J, Kim SK (2006). Chromosomal clustering and GATA transcriptional regulation of intestine-expressed genes in C. elegans. Development.

[ppat-0030199-b013] Yang Z, Edenberg HJ, Davis RL (2005). Isolation of mRNA from specific tissues of Drosophila by mRNA tagging. Nucl Acids Res.

[ppat-0030199-b014] Penalva L, Burdick M, Lin S, Sutterluety H, Keene J (2004). RNA-binding proteins to assess gene expression states of co-cultivated cells in response to tumor cells. Mol Cancer.

[ppat-0030199-b015] Griffin DE, Knipe DM, Fields BN, Howley PM (2001). Alphaviruses. Fields virology. 4th edition.

[ppat-0030199-b016] Anishchenko M, Bowen RA, Paessler S, Austgen L, Greene IP (2006). Venezuelan encephalitis emergence mediated by a phylogenetically predicted viral mutation. Proc Natl Acad Sci U S A.

[ppat-0030199-b017] Greene IP, Paessler S, Austgen L, Anishchenko M, Brault AC (2005). Envelope glycoprotein mutations mediate equine amplification and virulence of epizootic Venezuelan equine encephalitis virus. J Virol.

[ppat-0030199-b018] White LJ, Wang JG, Davis NL, Johnston RE (2001). Role of alpha/beta interferon in Venezuelan equine encephalitis virus pathogenesis: effect of an attenuating mutation in the 5' untranslated region. J Virol.

[ppat-0030199-b019] Powers AM, Brault AC, Kinney RM, Weaver SC (2000). The use of chimeric Venezuelan equine encephalitis viruses as an approach for the molecular identification of natural virulence determinants. J Virol.

[ppat-0030199-b020] MacDonald GH, Johnston RE (2000). Role of dendritic cell targeting in Venezuelan equine encephalitis virus pathogenesis. J Virol.

[ppat-0030199-b021] Bernard KA, Klimstra WB, Johnston RE (2000). Mutations in the E2 glycoprotein of Venezuelan equine encephalitis virus confer heparan sulfate interaction, low morbidity, and rapid clearance from blood of mice. Virology.

[ppat-0030199-b022] Aronson JF, Grieder FB, Davis NL, Charles PC, Knott T (2000). A single-site mutant and revertants arising in vivo define early steps in the pathogenesis of Venezuelan equine encephalitis virus. Virology.

[ppat-0030199-b023] Spotts DR, Reich RM, Kalkhan MA, Kinney RM, Roehrig JT (1998). Resistance to alpha/beta interferons correlates with the epizootic and virulence potential of Venezuelan equine encephalitis viruses and is determined by the 5' noncoding region and glycoproteins. J Virol.

[ppat-0030199-b024] Grieder FB, Nguyen HT (1996). Virulent and attenuated mutant Venezuelan equine encephalitis virus show marked differences in replication in infection in murine macrophages. Microb Pathog.

[ppat-0030199-b025] Grieder FB, Davis NL, Aronson JF, Charles PC, Sellon DC (1995). Specific restrictions in the progression of Venezuelan equine encephalitis virus-induced disease resulting from single amino acid changes in the glycoproteins. Virology.

[ppat-0030199-b026] Davis NL, Brown KW, Greenwald GF, Zajac AJ, Zacny VL (1995). Attenuated mutants of Venezuelan equine encephalitis virus containing lethal mutations in the PE2 Cleavage signal combined with a second-site suppressor mutation in E1. Virology.

[ppat-0030199-b027] Davis NL, Grieder FB, Smith JF, Greenwald GF, Valenski ML (1994). A molecular genetic approach to the study of Venezuelan equine encephalitis virus pathogenesis. Arch Virol Suppl.

[ppat-0030199-b028] Kinney RM, Chang GJ, Tsuchiya KR, Sneider JM, Roehrig JT (1993). Attenuation of Venezuelan equine encephalitis virus strain TC-83 is encoded by the 5'-noncoding region and the E2 envelope glycoprotein. J Virol.

[ppat-0030199-b029] Davis NL, Powell N, Greenwald GF, Willis LV, Johnson BJ (1991). Attenuating mutations in the E2 glycoprotein gene of Venezuelan equine encephalitis virus: construction of single and multiple mutants in a full-length cDNA clone. Virology.

[ppat-0030199-b030] Johnston RE, Smith JF (1988). Selection for accelerated penetration in cell culture coselects for attenuated mutants of Venezuelan equine encephalitis virus. Virology.

[ppat-0030199-b031] Johnson BJ, Kinney RM, Kost CL, Trent DW (1986). Molecular determinants of alphavirus neurovirulence: nucleotide and deduced protein sequence changes during attenuation of Venezuelan equine encephalitis virus. J Gen Virol.

[ppat-0030199-b032] Wang E, Brault AC, Powers AM, Kang W, Weaver SC (2003). Glycosaminoglycan binding properties of natural Venezuelan equine encephalitis virus isolates. J Virol.

[ppat-0030199-b033] Pushko P, Parker M, Ludwig GV, Davis NL, Johnston RE (1997). Replicon-helper systems from attenuated Venezuelan equine encephalitis virus: expression of heterologous genes in vitro and immunization against heterologous pathogens in vivo. Virology.

[ppat-0030199-b034] Baric RS, Carlin LJ, Johnston RE (1983). Requirement for host transcription in the replication of Sindbis virus. J Virol.

[ppat-0030199-b035] Moran TP, Collier M, McKinnon KP, Davis NL, Johnston RE (2005). A novel viral system for generating antigen-specific T cells. J Immunol.

[ppat-0030199-b036] Gardner JP, Frolov I, Perri S, Ji Y, MacKichan ML (2000). Infection of human dendritic cells by a Sindbis virus replicon vector is determined by a single amino acid substitution in the E2 glycoprotein. J Virol.

[ppat-0030199-b037] Ryman KD, Klimstra WB, Nguyen KB, Biron CA, Johnston RE (2000). Alpha/beta interferon protects adult mice from fatal Sindbis virus infection and is an important determinant of cell and tissue tropism. J Virol.

[ppat-0030199-b038] Klimstra WB, Nangle EM, Smith MS, Yurochko AD, Ryman KD (2003). DC-SIGN and L-sign can act as attachment receptors for alphaviruses and distinguish between mosquito cell- and mammalian cell-derived viruses. J Virol.

[ppat-0030199-b039] Ryman KD, Meier KC, Nangle EM, Ragsdale SL, Korneeva NL (2005). Sindbis virus translation is inhibited by a PKR/RNase L-independent effector induced by alpha/beta interferon priming of dendritic cells. J Virol.

[ppat-0030199-b040] Shabman RS, Morrison TE, Moore C, White L, Suthar MS (2007). Differential induction of type I interferon responses in myeloid dendritic cells by mosquito and mammalian-cell-derived alphaviruses. J Virol.

[ppat-0030199-b041] Jordan GW (1973). Interferon sensitivity of Venezuelan equine encephalomyelitis virus. Infect Immun.

[ppat-0030199-b042] Jahrling PB, Navarro E, Scherer WF (1976). Interferon induction and sensitivity as correlates to virulence of Venezuelan encephalitis viruses for hamsters. Arch Virol.

[ppat-0030199-b043] Swanson KA, Zheng Y, Heidler KM, Zhang Z-D, Webb TJ (2004). Flt3-Ligand, IL-4, GM-CSF, and adherence-mediated isolation of murine lung dendritic cells: assessment of isolation technique on phenotype and function. J Immunol.

[ppat-0030199-b044] Shanmugam N, Ransohoff RM, Natarajan R (2006). Interferon-gamma-inducible protein (IP)-10 mRNA stabilized by RNA-binding proteins in monocytes treated with S100b. J Biol Chem.

[ppat-0030199-b045] Mangus D, Evans M, Jacobson A (2003). Poly(A)-binding proteins: multifunctional scaffolds for the post-transcriptional control of gene expression. Genome Biol.

[ppat-0030199-b046] Grieder FB, Davis BK, Zhou XD, Chen SJ, Finkelman FD (1997). Kinetics of cytokine expression and regulation of host protection following infection with molecularly cloned Venezuelan equine encephalitis virus. Virology.

[ppat-0030199-b047] Anishchenko M, Paessler S, Greene IP, Aguilar PV, Carrara AS (2004). Generation and characterization of closely related epizootic and enzootic infectious cDNA clones for studying interferon sensitivity and emergence mechanisms of Venezuelan equine encephalitis virus. J Virol.

[ppat-0030199-b048] Schoneboom BA, Lee JS, Grieder FB (2000). Early expression of IFN-alpha/beta and iNOS in the brains of Venezuelan equine encephalitis virus-infected mice. J Interferon Cytokine Res.

[ppat-0030199-b049] Grieder FB, Vogel SN (1999). Role of interferon and interferon regulatory factors in early protection against Venezuelan equine encephalitis virus infection. Virology.

[ppat-0030199-b050] Charles PC, Trgovcich J, Davis NL, Johnston RE (2001). Immunopathogenesis and immune modulation of Venezuelan equine encephalitis virus-induced disease in the mouse. Virology.

[ppat-0030199-b051] Schoneboom BA, Catlin KM, Marty AM, Grieder FB (2000). Inflammation is a component of neurodegeneration in response to Venezuelan equine encephalitis virus infection in mice. J Neuroimmunol.

[ppat-0030199-b052] Jackson AC, John PR (1997). Apoptotic cell death is an important cause of neuronal injury in experimental Venezuelan equine encephalitis virus infection of mice. Acta Neuropathologica.

[ppat-0030199-b053] Darman J, Backovic S, Dike S, Maragakis NJ, Krishnan C (2004). Viral-induced spinal motor neuron death is non-cell-autonomous and involves glutamate excitotoxicity. J Neurosci.

[ppat-0030199-b054] Carmen J, Genevieve Gowing Jean-Pierre Julien Douglas Kerr (2006). Altered immune response to CNS viral infection in mice with a conditional knock-down of macrophage-lineage cells. Glia.

[ppat-0030199-b055] Thompson JM, Whitmore AC, Konopka JL, Collier ML, Richmond EMB (2006). Mucosal and systemic adjuvant activity of alphavirus replicon particles. Proc Natl Acad Sci U S A.

[ppat-0030199-b056] Serody JS, Collins EJ, Tisch RM, Kuhns JJ, Frelinger JA (2000). T Cell activity after dendritic cell vaccination is dependent on both the type of antigen and the mode of delivery. J Immunol.

