# Supplementary material for: A Two-Phase Innate Host Response to Alphavirus Infection Identified by mRNP-Tagging In Vivo
Source: PLoS Pathog. 2007 Dec 21;3(12):e199. doi: 10.1371/journal.ppat.0030199 (PMC2151086; doi:10.1371/journal.ppat.0030199)
Supplement: Supplementary file 1 [file ppat.0030199.sg001.pdf]

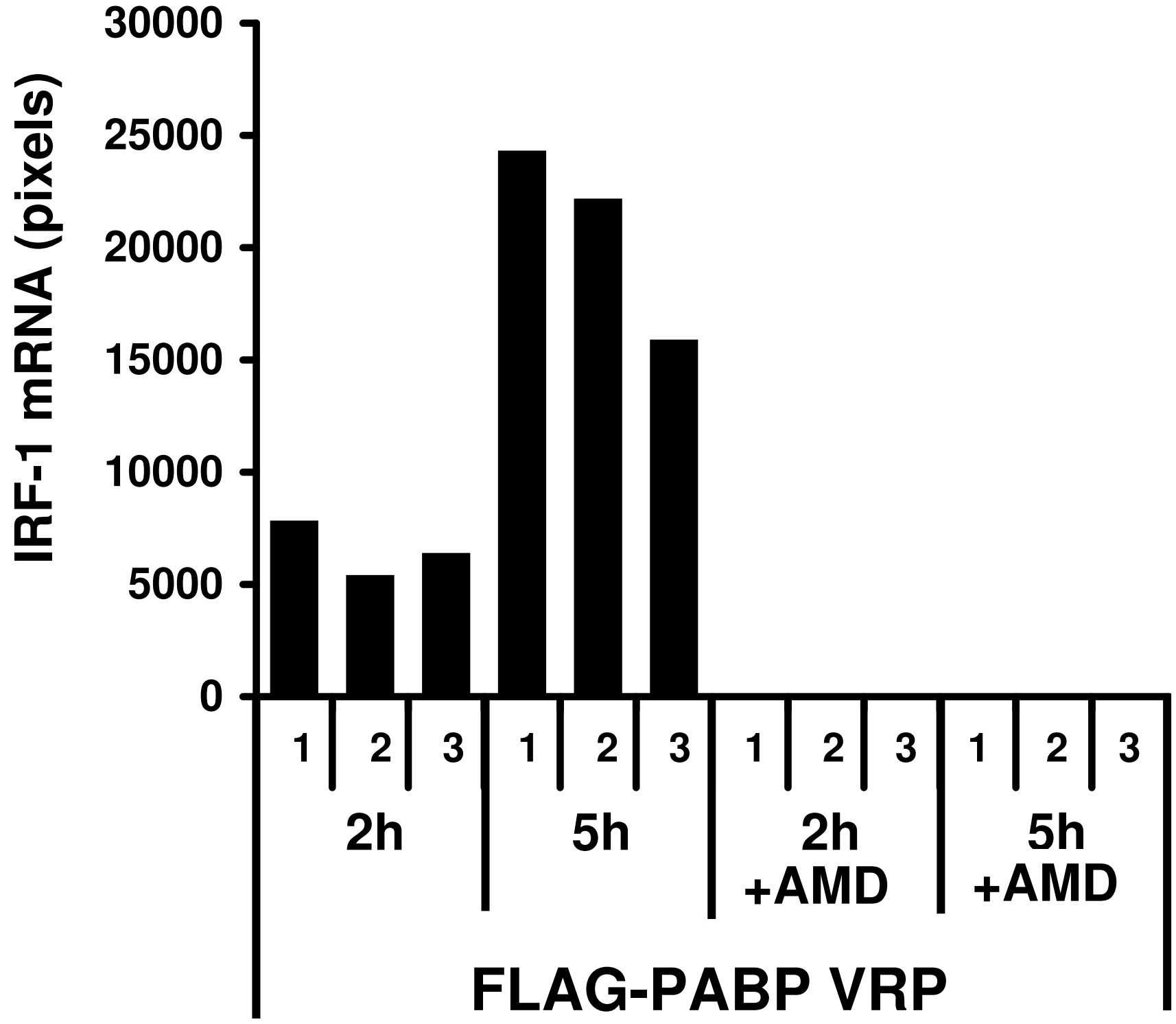

IRF-1 mRNA (pixels)
30000
25000
20000
15000
10000
5000
0
1
2
3
1
2
3
1
2
3
1
2
3
2h
5h
2h +AMD
5h +AMD
FLAG-PABP VRP
