# Supplementary material for: A Two-Phase Innate Host Response to Alphavirus Infection Identified by mRNP-Tagging In Vivo
Source: PLoS Pathog. 2007 Dec 21;3(12):e199. doi: 10.1371/journal.ppat.0030199 (PMC2151086; doi:10.1371/journal.ppat.0030199)
Supplement: Supplementary file 2 [file ppat.0030199.sg002.pdf]

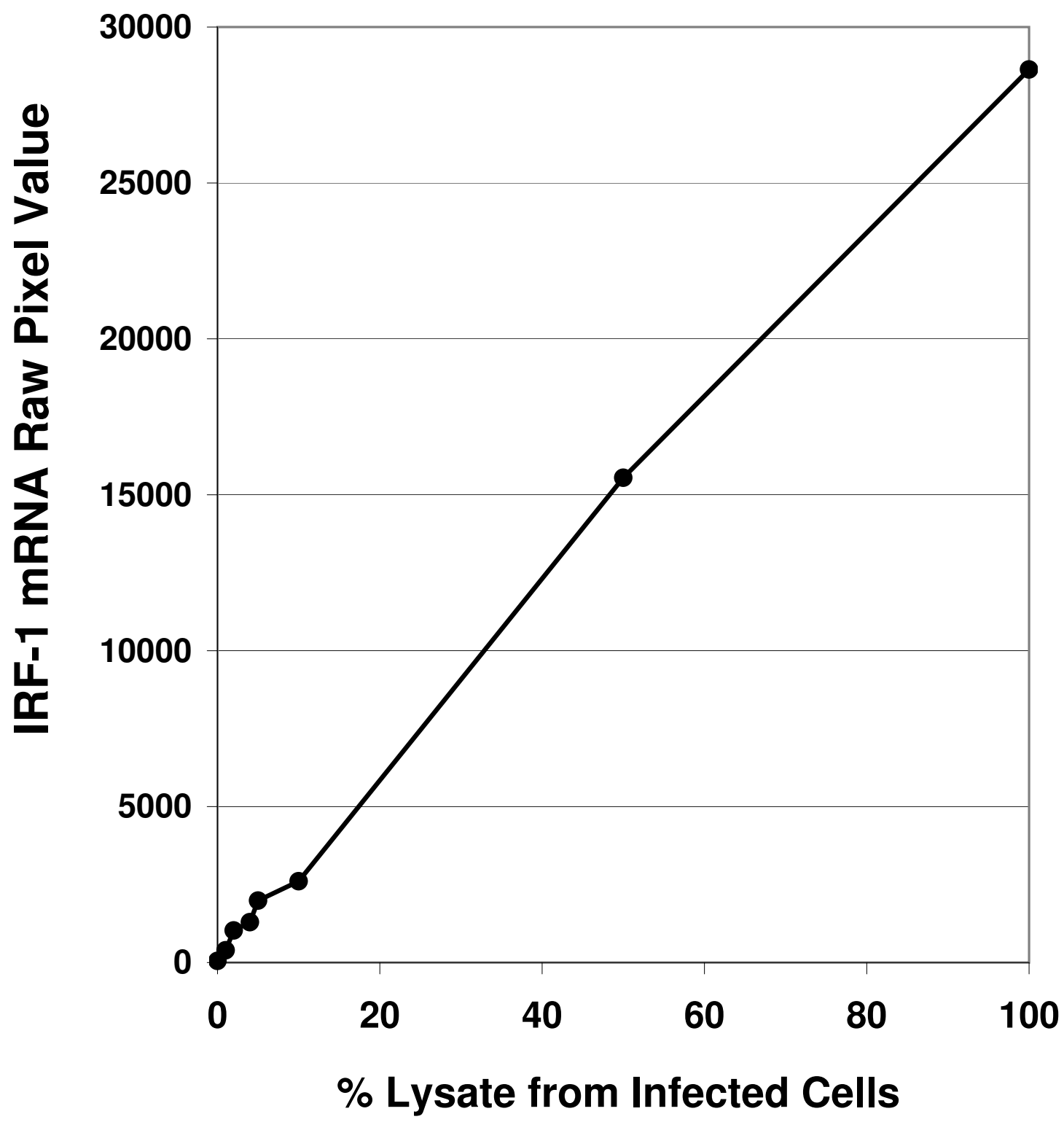

30000
25000
20000
15000
10000
5000
0
0
20
40
60
80
100
IRF-1 mRNA Raw Pixel Value
% Lysate from Infected Cells
