# Supplementary material for: A Two-Phase Innate Host Response to Alphavirus Infection Identified by mRNP-Tagging In Vivo
Source: PLoS Pathog. 2007 Dec 21;3(12):e199. doi: 10.1371/journal.ppat.0030199 (PMC2151086; doi:10.1371/journal.ppat.0030199)
Supplement: Supplementary file 3 [file ppat.0030199.sg003.pdf]

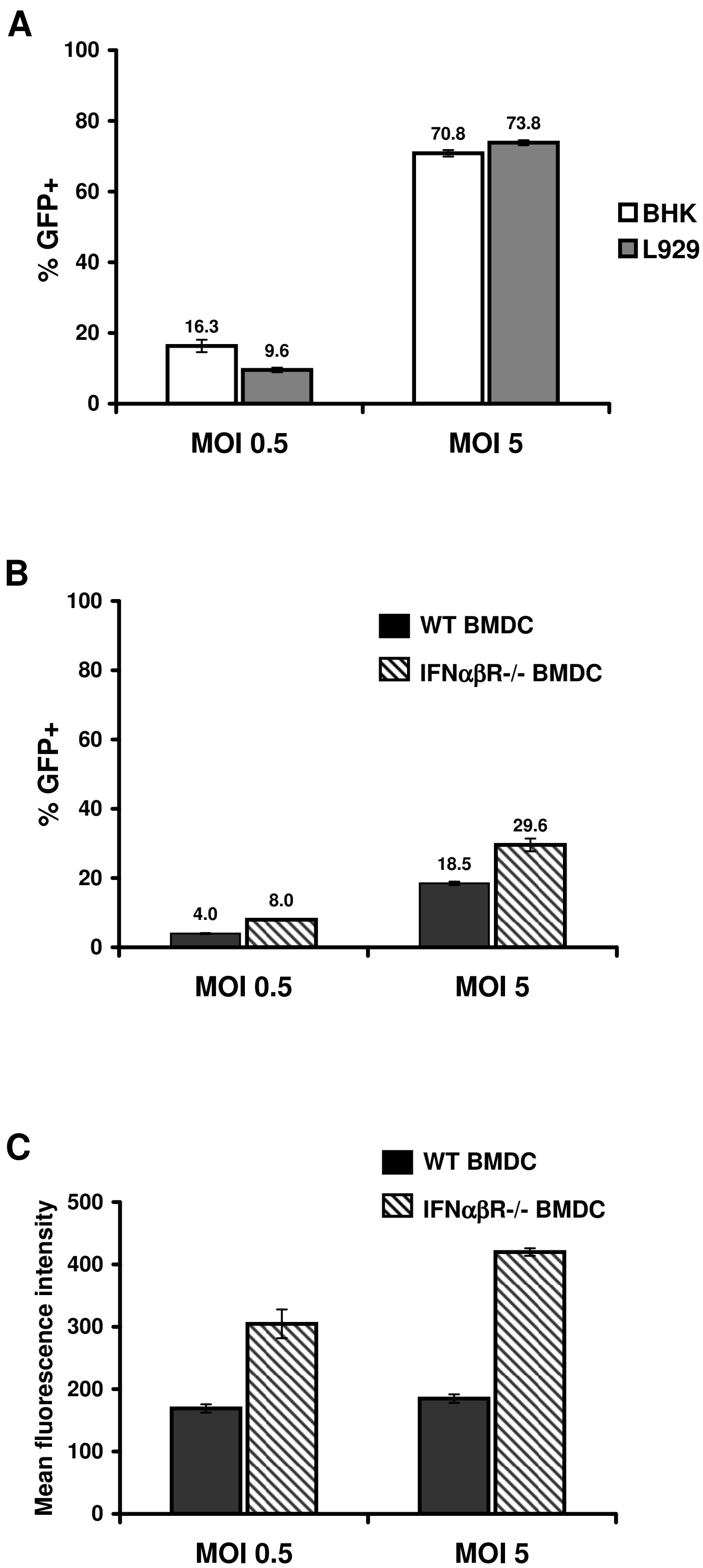
A
100
80
60
40
20
0
% GFP+
16.3
9.6
70.8
73.8
BHK
L929
MOI 0.5
MOI 5
B
100
80
60
40
20
0
% GFP+
WT BMDC
IFNαβR-/- BMDC
4.0
8.0
18.5
29.6
MOI 0.5
MOI 5
C
WT BMDC
IFNαβR-/- BMDC
500
400
300
200
100
0
Mean fluorescence intensity
MOI 0.5
MOI 5
